# *cis*-Clerodane-type diterpenoids from *Tinospora crispa* and their anticancer potential

**DOI:** 10.1007/s12272-026-01596-y

**Published:** 2026-01-20

**Authors:** Se Yun Jeong, Jisun Kim, Ji Won Ha, Norhayati Ahmad, Nurul Hazlina Zaini, Yoon-Joo Ko, Alan Jung Park, Wonhwa Lee, Ki Hyun Kim

**Affiliations:** 1 School of Pharmacy, Sungkyunkwan University, Suwon, 16419 Republic of Korea; 2 Department of Chemistry, Sungkyunkwan University, Suwon, 16419 Republic of Korea; 3 Institute for Biodiversity and Environmental Research, Universiti Brunei Darussalam, Jalan Tunku Link, Gadong, BE1410 Brunei Darussalam; 4 Universiti Brunei Darussalam Botanical Research Centre, Institute for Biodiversity and Environmental Research, Universiti Brunei Darussalam, Jalan Tunku Link, Gadong, BE1410 Brunei Darussalam; 5 Laboratory of Nuclear Magnetic Resonance, National Center for Inter-University Research Facilities (NCIRF), Seoul National University, Seoul, 08826 Republic of Korea; 6 Department of Physiology, Seoul National University College of Medicine, Seoul, Republic of Korea

**Keywords:** *Tinospora crispa*, *cis*-Clerodane-type diterpenoids, PANIC, Anticancer, LLC1, A549

## Abstract

*Tinospora crispa* (Menispermaceae) has been traditionally consumed as a functional food and herbal remedy in Southeast Asia, notably in Thailand and India. *cis*-Clerodane-type diterpenoids represent the characteristic and predominant metabolites of the genus *Tinospora*. Chemical investigation of a MeOH extract of *T. crispa* leaves, guided by LC/MS analysis coupled with an in-house UV spectral library, led to the isolation of five compounds (**1**–**5**), including four new *cis*-clerodane-type diterpenoids (**1**–**4**). Their structures were elucidated by 1D and 2D NMR spectroscopy, high-resolution mass spectrometry (HR-ESIMS), interproton distance analysis using NOE peak amplitude normalization for improved cross-relaxation (PANIC), Snatzke’s method, and computational ECD and DP4⁺ probability calculations. The isolated compounds (**1**–**5**) were evaluated for their anticancer potential in both liver (Hepa1c1c7, Hepa1-6) and lung (LLC1, A549) cancer cell lines. All compounds **1**–**5** reduced A549 cell viability by approximately 70%, at 200 μM and showing comparable activity in LLC1. Molecular analyses showed that compound **3** affected downstream Hippo signaling components (YAP, TAZ, pan-TEAD) in liver cancer cells and inhibited pro-survival pathways—including phosphorylated AKT—in lung cancer cells, where it also elevated apoptotic markers Bax and cleaved caspase-3 while reducing anti-apoptotic BCL-2. Overall, compound **3** exhibited the most consistent and potent cell-line specific anticancer effects across both models, highlighting its potential as a promising lead candidate for further anticancer drug development. Collectively, these results suggest concentration-dependent anticancer activity of *T. crispa* diterpenoids in liver and lung cancer models and further support compound **3** as promising leading candidate targeting key survival signaling pathways in cancer.

**Supplementary Information:**

The online version contains supplementary material available at 10.1007/s12272-026-01596-y.

## Introduction

*Tinospora crispa* (Menispermaceae), commonly known as Petawali, is a climbing plant found in subtropical regions of Southeast Asia and Africa, including Thailand, Malaysia, and Indonesia (Ahmad et al. 2016; Hossen et al. 2016). The stems of *T. crispa* are long, woody vines (4–20 m in length) with characteristic protuberances, and the large, heart-shaped leaves measure approximately 6–12 cm in length and 7–12 cm in width (Ahmad et al. 2016). *T. crispa* fruit has been traditionally consumed as food in India, and notably in Thailand, its seeds are cold-infused in water and used as a detoxifying remedy for alcohol poisoning (Srithi et al. 2009; French et al. 2022). Phy-Blica-O is a Thai polyherbal formula based primarily on *Phyllanthus emblica* (Indian gooseberry) and includes components from 11 different medicinal plants such as garlic and ginger, among others. Importantly, *T. crispa* is one of the key ingredients in Phy-Blica-O, contributing a strong bitter taste to the herbal tea. This tea has long been traditionally enjoyed by Thai people as a rejuvenating and health-promoting beverage (Issuriya et al. 2019). *T. crispa* is widely consumed in traditional diets as a functional food due to its reputed health benefits, particularly for the management of type 2 diabetes mellitus (Noor et al. 1998; Thomas et al. 2016; Klangjareonchai et al. 2012). Species of *Tinospora* are recognized in Ayurvedic traditions as valuable functional herbs owing to their broad spectrum of beneficial properties, including antidiabetic, antiallergic, anti-inflammatory, antioxidant, antispasmodic, hepatoprotective, and immunomodulatory effects. Additionally, *T. crispa* has been traditionally consumed in Thailand, Malaysia, and Indonesia as a functional food for promoting health, including reducing internal inflammation, supporting blood glucose control, managing hypertension, stimulating appetite, and even offering protection against mosquito bites (Chakraborty et al. 2020; Shul′ ts et al. 2014).

The stem extract of *T. crispa* demonstrates notably high antioxidant activity in assays such as DPPH radical scavenging, reducing power, and metal chelating capacity (ZulkEfli et al. 2013). In an alloxan-induced diabetic male Wistar albino rat model, administration of aqueous extract of *T. crispa* at a concentration of 4 g/L in drinking water resulted in lower fasting blood glucose levels and increased serum insulin levels compared with untreated diabetic controls (Noor et al. 1989). Extensive studies have demonstrated the anti-tumor activity of *T. crispa*. For example, the aqueous extract of *T. crispa* exhibits moderate anti-proliferative effects against various human cancer cell lines, with IC_50_ values of approximately 107 μg/mL for MCF-7, 165 μg/mL for HeLa, 100 μg/mL for Caov-3, and 165 μg/mL for HepG2 cells (Zulkhairi et al. 2008). Notably, *T. crispa* shows pronounced anti-proliferative activity against triple-negative breast cancer cells, inducing apoptosis in approximately 92% of treated cells and exerting synergistic effects when combined with cisplatin through significant downregulation of NF-κB gene expression (Al-Rashidi et al. 2016). Beyond breast cancer, *T. crispa* has also demonstrated anticancer potential in head and neck squamous cell carcinoma (HNSCC). Treatment with *T. crispa* extract at 100 mg/mL reduced cell viability to approximately 50–60% in metastatic HNSCC cell lines (HN22 and HSC-3), while non-cytotoxic concentrations dose-dependently suppressed MMP-13 expression and inhibited cell migration by up to 65%, indicating notable anti-metastatic properties (Phienwej et al. 2015). In addition, tetrandrine and other alkaloids isolated from *T. crispa* have shown potent anticancer activities; notably, tetrandrine has been reported to reverse multidrug resistance in cancer cell lines by downregulating the expression of the ABCB1 transporter (Rasad et al., 2023).

Previous phytochemical investigations have revealed that *T. crispa* contains diterpenoids, steroids, flavonoids, lignans, alkaloids, and phenolic compounds (Choudhary et al. 2010; Li et al. 2016; Koay et al. 2013). Among these, *cis*-clerodane-type diterpenoids are considered characteristic and major constituents of the genus *Tinospora*. These compounds have been reported to exhibit antidiabetic and hypoglycemic effects, including significant reductions in serum glucose levels in hyperglycemic mice (Ruan et al. 2012; Lam et al. 2012). Clerodane-type diterpenoids have also demonstrated significant anticancer activities against both liver and lung cancers by inducing apoptosis, inhibiting cancer cell proliferation, and disrupting key signaling pathways, underscoring their potential as promising natural therapeutic agents for these malignancies (Dai et al. 2022; Tatipamula et al. 2022; Ma et al. 2019; Liu et al. 2020). For instance, mallotucin D, isolated from *Croton crassifolius*, along with other clerodane diterpenes containing a *γ*-butyrolactone moiety from *Polyalthia longifolia*, have shown potent inhibitory effects on the growth of hepatocellular carcinoma cells (HepG2) (Dai et al. 2022; Tatipamula et al. 2022). Additionally, clerodane-type diterpenoids such as kurzipenes from *Casearia kurzii* and graveospenes from *Casearia graveolens*, which possess acetyloxy groups, have exhibited strong anticancer activity against human lung cancer cell lines (A549) (Ma et al. 2019; Liu et al. 2020). However, despite these findings, comprehensive mechanistic investigations focusing on *cis*-clerodane diterpenoids isolated from *T. crispa*, particularly regarding their cellular effects in hepatic and pulmonary cancer models, remain limited. This gap underscores the need for further studies to elucidate their underlying signaling pathway modulation.

As part of our ongoing efforts to discover biologically active compounds from diverse resources (Lee et al. 2024; Bridget et al. 2025; Gil et al. 2024; Cho et al. 2024; Kim et al. 2025a; Glick et al. 2024), we conducted a chemical analysis of the MeOH extract of *T. crispa* leaves, guided by LC/MS profiling combined with an in-house UV spectral library, which led to the isolation of five compounds (**1**–**5**), including four new *cis*-clerodane-type diterpenoids (**1**–**4**). The structures of the new compounds were elucidated by extensive spectroscopic analysis, including 1D and 2D NMR and high-resolution mass spectrometry (HR-ESIMS). Their absolute configurations were established through NOESY analysis, interproton distance analysis via the PANIC (peak amplitude normalization for improved cross-relaxation) method, Snatzke’s method, and computational ECD and DP4 + probability calculations. Herein, we present the isolation and structural elucidation of compounds **1**–**5**, along with an evaluation of the anticancer potential of the isolated compounds considering the well-documented anticancer activity of clerodane-type diterpenoids.

## Materials and methods

### General experimental procedures

Optical rotations were measured on a Jasco P-2000 polarimeter (Jasco, Easton, MD, USA). Ultraviolet (UV) spectra were acquired on an Agilent 8453 UV–visible spectrophotometer (Agilent Technologies, Santa Clara, CA, USA). Electronic circular dichroism (ECD) spectra were obtained on a Jasco J-1500 spectropolarimeter (Jasco, Easton, MD, USA). NMR spectra were recorded on a Bruker AVANCE III HD 850 NMR spectrometer (Bruker, Billerica, MA, USA) with a 5 mm TCI CryoProbe, operating at 850 MHz (^1^H) and 212.5 MHz (^13^C). Chemical shifts are reported in ppm (*δ*) for ^1^H and ^13^C NMR, referenced to solvent peaks of CD_3_OD (Cambridge Isotope Laboratories, Inc.) at 3.310 ppm for ^1^H and 49.000 ppm for ^13^C. LC/MS analyses were carried out on an Agilent 1200 Series HPLC system equipped with a diode array detector and a 6130 Series ESI mass spectrometer, using a Kinetex C18 100 Å column (100 × 2.1 mm, 5 μm; flow rate: 0.3 mL/min; Phenomenex). High-resolution electrospray ionization mass spectrometry (HR-ESIMS) data were acquired on an Agilent 6545 quadrupole time-of-flight (Q-TOF) LC/MS system (Agilent Technologies). Preparative and semi-preparative HPLC were performed using a Waters 1525 binary HPLC pump with a Waters 996 photodiode array detector (Waters Corp., Milford, MA, USA) on an Agilent Eclipse C18 column (250 × 21.2 mm, 5 μm; flow rate: 5 mL/min) and a Phenomenex Luna Phenyl-Hexyl 100 Å column (250 × 10 mm, 5 μm; flow rate: 2 mL/min; Phenomenex, Torrance, CA, USA), respectively. Column chromatography was carried out using silica gel 60 (230–400 mesh; Merck, Darmstadt, Germany) and RP-C18 silica gel (230–400 mesh; Merck). Thin-layer chromatography (TLC) was performed on precoated silica gel 60 F₂₅₄ and RP-C18 F₂₅₄s plates (Merck), and spots were visualized under UV light or by heating after spraying with anisaldehyde–sulfuric acid reagent.

### Chemicals

All organic solvents used in this study including *n*-hexane, dichloromethane, ethyl acetate, *n*-butanol, acetone, acetonitrile (MeCN), and methanol (MeOH) were of analytical grade and were purchased from Samchun Chemical Co., Ltd. (Seoul, Korea). NMR solvents were obtained from Cambridge Isotope Laboratories (Tewksbury, MA, USA). For LC/MS analysis, MeCN, MeOH, water, and formic acid (purity ≥ 98.0%) were LC/MS-grade and were obtained from Fisher Scientific (Ottawa, ON, Canada).

### Plant material

The leaves of *T. crispa* were collected from the Botanical Research Centre, Universiti Brunei Darussalam, and were received in extracted form by Dr. Norhayati Ahmad. The plant was identified by a Botanist, Nurul Hazlina Zaini at the Botanical Research Centre. A voucher specimen (UBDBRC2020-0001/IBER-H 000 740) has been deposited in the Institute for Biodiversity and Environmental Research Herbarium (IBER herbarium).

### Extraction and isolation of compound

Leaves of *T. crispa* (100 g) were collected, shade-dried at room temperature (22.4 ± 2.0 °C), and ground using an electric grinding mill (IKA®-WERKE MF 10, IKA-Werke GmbH & Co. KG, Staufen, Germany). The powdered material was sieved and extracted by Soxhlet extraction using MeOH. The solvent was removed under reduced pressure using a rotary evaporator to yield a crude methanol extract (10.3 g). This extract was suspended in distilled water (700 mL) and successively partitioned with *n*-hexane, dichloromethane (CH₂Cl₂), ethyl acetate (EtOAc), and *n*-BuOH to afford four fractions of increasing polarity: *n*-hexane-soluble (1.7 g), CH₂Cl₂-soluble (0.6 g), EtOAc-soluble (0.3 g), and *n*-BuOH-soluble (1.0 g) fractions. LC/MS analysis of each fraction, guided by an in-house UV spectral library, revealed that diterpenoids were primarily enriched in the CH₂Cl₂- and *n*-BuOH-soluble fractions. The CH₂Cl₂-soluble fraction (0.6 g) was subjected to silica gel column chromatography using a gradient solvent system (CH₂Cl₂/MeOH, 90:1 → 5:1, v/v), affording five subfractions (C1–C5). Fraction C4 (163.9 mg) was further fractionated by reversed-phase silica gel column chromatography using a gradient solvent system (40% MeOH/H₂O → 100% MeOH), yielding four subfractions (C41–C44). Subfraction C43 was purified by semi-preparative reversed-phase HPLC (50% MeOH/H₂O) to afford compounds **1** (*t*_R_ = 30.2 min, 5.0 mg) and **3** (*t*_R_ = 71.0 min, 4.9 mg). Fraction C44 (38.9 mg) was purified using semi-preparative reversed-phase HPLC (57% MeOH/H₂O) to yield compound **4** (*t*_R_ = 91.0 min, 0.6 mg). The *n*-BuOH-soluble fraction (1.0 g) was subjected to reversed-phase silica gel column chromatography using a gradient solvent system (50% MeOH/H₂O → 100% MeOH), affording seven subfractions (B1–B7). Fraction B3 (309.1 mg) was further separated by preparative reversed-phase HPLC using a gradient solvent system (30% MeOH/H₂O → 50% MeOH/H₂O), yielding six subfractions (B31–B36). Subfraction B31 (57.1 mg) was purified by semi-preparative reversed-phase HPLC (18% MeOH/H₂O) to afford compound **5** (*t*_R_ = 55.6 min, 1.3 mg). Fraction B5 (93.1 mg) was further separated on a Sephadex LH-20 column using CH₂Cl₂/MeOH (2:8, v/v), affording five subfractions (B51–B55). Subfraction B53 (57.1 mg) was purified by semi-preparative reversed-phase HPLC (35% MeOH/H₂O) to yield compound **2** (*t*_R_ = 62.5 min, 3.6 mg).

### Tinospordane A (1)

Colorless gum; ${[\alpha ]}_{\mathrm{D}}^{20}$ +3.8 (*c* 0.25, MeOH); UV (MeOH) λ_max_ (log ε) 206 (2.4) nm; ECD (3.1 mg/mL, MeOH) λ_max_ (Δ*ε*) 218 (-0.08), 233 (+ 1.73) nm; ^1^H (850 MHz) and ^13^C (212.5 MHz) NMR data (Table 1); HR-ESIMS (negative-ion mode) *m/z* 361.1665 [M − H]^−^ (calcd for C_20_H_25_O_6_, 361.1657).

**Table 1:** ^1^H (850 MHz) and ^13^C NMR (212.5 MHz) data of compounds **1**–**4** in CD_3_OD (*δ* ppm)

Position	**1**		**2**		**3**		**4**	
	*δ*_C_, type	*δ*_H_ *(J* in Hz)	*δ*_C_^*a*^, type	*δ*_H_ *(J* in Hz)	*δ*_C_^*a*^, type	*δ*_H_ *(J* in Hz)	*δ*_C_, type	*δ*_H_ *(J* in Hz)
1	24.2, CH_2_	*α* 2.36 ddt (15.5, 9.5, 3.0) *β* 2.22 m	23.8, CH_2_	*α* 2.35 dddd (16.0, 9.5, 6.5, 3.0) *β* 2.18 m	21.9, CH_2_	*α* 1.89 m *β* 1.58 m	26.2, CH_2_	*α* 2.26 m *β* 2.21 m
2	135.5, CH	6.26 ddd (9.5, 6.5, 3.0)	135.8, CH	6.27 ddd (9.5, 6.5, 3.0)	29.8, CH_2_	*α* 1.93 m *β* 1.76 m	131.8, CH	5.97 dtd (9.5, 4.5, 2.5)
3	126.8, CH	5.77 dd (9.5, 3.0)	126.6, CH	5.75 dd (9.5, 3.0)	73.8, CH	3.81 dd (5.0, 3.5)	129.4, CH	5.65 dt (10.0, 2.0)
4	78.3, C		78.3, C		82.7, C		79.7, C	
5	47.5, C		47.3, C		43.7, C		43.0, C	
6	78.1, CH	4.58 dd (12.0, 7.5)	78.2, CH	4.58 dd (12.0, 7.5)	28.0, CH_2_	*α* 1.25 m *β* 2.51 m	30.5, CH_2_	*α* 1.15 dt (14.0, 7.5) *β* 1.96 dt (14.0, 7.0)
7	29.6, CH_2_	*α* 2.07 ddd (13.0, 12.0, 8.5) *β* 1.71 dt (13.0, 7.5)	29.5, CH_2_	*α* 2.02 m *β* 1.67 m	27.5, CH_2_	*α* 1.27 m *β* 1.73 m	28.3, CH_2_	*α* 1.27 m *β* 1.60 m
8	33.7, CH	2.20 m	33.0, CH_2_	2.19 m	37.5, CH	1.61 m	35.2, CH	1.79 m
9	41.4, C		41.0, C		40.3, C		40.0, C	
10	41.1, CH	2.05 dd (9.5, 2.5)	40.6, CH	2.08 ddd (9.5, 7.5, 2.0)	44.0, CH	1.75 dd (10.5, 4.5)	42.8, CH	1.92 t (7.0)
11	38.9, CH_2_	a 1.58 m b 1.67 m	34.3, CH_2_	1.39 m	41.1, CH_2_	a 1.55 m b 1.95 m	39.8, CH_2_	1.62 m
12	23.2, CH_2_	2.39 m	28.1, CH_2_	a 1.49 m b 1.59 m	24.0, CH_2_	2.40 m	20.6, CH_2_	a 2.15 m b 2.22 m
13	172.3, C		76.6, C		171.4, C		140.1, C	
14	117.8, CH	5.93 s	76.0, CH	3.63 m	117.4, CH	5.87 s	47.8, CH_2_	3.92 q (2.0)
15	173.6, C		63.4, CH	a 3.62 m b 3.76 m	173.8, C		176.9, C	
16	101.0, CH	6.06 br s	64.5, CH	3.53 m	101.3, CH	6.03 br s	139.6, CH	6.89 p (2.0)
17	17.5, CH_3_	1.02 d (7.0)	17.3, CH_3_	1.00 d (7.0)	18.2, CH_3_	0.98 d (7.0)	17.2, CH_3_	0.86 d (7.0)
18	180.7, C		181.0, C		176.9, C		176.9, C	
19	18.3, CH_3_	1.23 s	18.1, CH_3_	1.21 s	27.4, CH_3_	1.23 s	28.5, CH_3_	1.25 s
20	20.6, CH_3_	0.95 s	20.6, CH_3_	0.89 s	22.6, CH_3_	0.92 s	19.4, CH_3_	0.79 s
18-OCH_3_					53.0, CH_3_	3.76 s	52.4, CH_3_	3.70 s

^*a*^^13^C NMR data were measured and assigned based on the HSQC and HMBC experiments

### Tinospordane B (2)

Colorless gum; ${[\alpha ]}_{\mathrm{D}}^{20}$ +13.6 (*c* 0.18, MeOH); UV (MeOH) λ_max_ (log ε) 200 (1.2) nm; ECD (3.0 mg/mL, MeOH) λ_max_ (Δ*ε*) 207 (-0.48), 231 (+ 0.29) nm; ^1^H (850 MHz) and ^13^C (212.5 MHz) NMR data (Table 1); HR-ESIMS (negative-ion mode) *m/z* 383.2072 [M − H]^−^ (calcd for C_20_H_31_O_7_, 383.2075).

### Tinospordane C (3)

Colorless gum; ${[\alpha ]}_{\mathrm{D}}^{20}$ +3.5 (*c* 0.25, MeOH); UV (MeOH) λmax (log ε) 206 (2.7) nm; ECD (4.0 mg/mL, MeOH) λmax (Δ*ε*) 218 (-0.05) nm, 229 (+ 0.11) nm; ^1^H (850 MHz) and ^13^C (212.5 MHz) NMR data (Table 1); HR-ESIMS (negative-ion mode) *m/z* 395.2087 [M − H]^−^ (calcd for C_21_H_31_O_7_, 395.2075).

### Tinospordane D (4)

Colorless gum; ${[\alpha ]}_{\mathrm{D}}^{20}$ +45.6 (*c* 0.03, MeOH); UV (MeOH) λ_max_ (log ε) 200 (2.1) nm; ECD (1.5 mg/mL, MeOH) λ_max_ (Δ*ε*) 200 (-0.09), 224 (+ 0.15), 246 (-0.08) nm; ^1^H (850 MHz) and ^13^C (212.5 MHz) NMR data (Table 1); HR-ESIMS (positive-ion mode) *m/z* 363.2166 [M + H]^+^ (calcd for C_21_H_31_O_5_, 363.2166).

### Computational analysis

Initial conformational searches were conducted using the OPLS4 force field in MacroModel (version 2024–4, Schrödinger LLC, New York, NY, USA) with mixed torsional/low-mode sampling. Searches were performed in the gas phase with an energy window of 20 kJ mol⁻^1^ and a maximum of 10,000 steps. Geometry minimization was carried out using the Polak-Ribiere conjugate gradient (PRCG) algorithm with a maximum of 10,000 iterations and a convergence threshold of 0.001 kJ·mol⁻^1^·Å⁻^1^ for the RMS gradient. Conformers within 10 kJ mol⁻^1^ were selected and optimized at the DFT B3LYP/6–31 + G(d,p) level using TmoleX 4.3.2.

DP4 + analysis was conducted by calculating the NMR shielding constants for 16*R*-**1**/16*S*-**1**, 13*R*-**2**/13*S*-**2**, and 16*R*-**3**/16*S*-**3** at the same level of theory and basis set (B3LYP/6–31 + G(d,p)) as employed in the ECD calculations. Chemical shifts (${\delta }_{calc}^{x}$) were calculated from magnetic shielding tensors using the equation:

$$\delta_{calc}^{x} = \sigma^{o} - \sigma^{x}$$

where ${\sigma }^{x}$ is the Boltzmann-averaged shielding constant for nucleus x, and ${\sigma }^{o}$ is the calculated shielding of TMS at the same level of theory. The calculated *δ* values were then compared with experimental data using the DP4 + probability method, implemented via the Excel tool (Grimblat et al. 2015).

ECD calculations (rotatory strength) were performed on **1A**/**1B** (14 conformers), **2A**/**2B** (19 conformers), **3A**/**3B** (13 conformers), and **4A**/**4B** (14 conformers). Calculated rotatory strength values were converted to ECD spectra using a Gaussian band-shape function with a bandwidth (*σ*) of 0.2 eV. The ECD intensity (Δ*ε*) was calculated using the equation:

$$\Delta \varepsilon \left( E \right) = \frac{1}{{2.297 \times 10^{ - 39} }}\frac{1}{{\sqrt {2\pi \sigma } }}\mathop \sum \limits_{A}^{i} \Delta E_{i} R_{i} e^{{\left[ { - \left( {E - \Delta E_{i} } \right)^{2} /\left( {2\sigma } \right)^{2} } \right]}}$$

where Δ*E*_*i*_ and *R*_*i*_ are the excitation energy and rotatory strength of transition *i*, respectively. The final ECD spectra were generated by Boltzmann weighting over all conformers and visualized using SigmaPlot 14.0 (Jeong et al. 2023).

### PANIC analysis

The PANIC analysis was carried out based on a previously reported method, with slight modifications (Lee et al. 2022). Two-dimensional NOESY spectrum slices of compounds **1** and **2** were acquired using MestReNova software (version 14.2.1–27,684, Mestrelab Research S.L., Santiago de Compostela, Spain). As a reference, the interproton distance between two *cis*-related olefinic protons, H-2 and H-3, was set to 2.5 Å (*r*_reference_). The corresponding NOE intensity (NOE_reference_) was obtained by integrating the H-3 signal in the slice irradiated at H-2. The NOE intensities (NOE_unknown_) for H-20 were acquired by irradiating H-6, followed by integration of the H-20 signals.

Calibrated interproton distances (*r*_unknwon_) were calculated using the following equation:

$$\frac{{NOE_{unknown} }}{{NOE_{reference} }} = \left( {\frac{{r_{reference} }}{{r_{unknown} }}} \right)^{6}$$

### ***ECD measurement of Mo***_***2***_***(OAc)***_***4***_*** complex (Snatzke’s method)***

A mixture of compound **2** (0.5 mg) and Mo₂(OAc)₄ (0.6 mg) in 0.9 mL of DMSO was prepared at a ligand-to-metal ratio of approximately 1:1, and the resulting solution was directly subjected to ECD measurements. The initial ECD spectrum was recorded immediately after mixing, and time-dependent spectral changes were monitored until a stable spectrum was observed (approximately 30 min after mixing). The inherent ECD spectrum of compound **2** was subtracted. The observed sign of the diagnostic band at approximately 310 nm in the induced ECD spectrum was used to determine the absolute configuration of the 1,2-diol moiety (Di Bari et al. 2001).

### Cell culture and reagents

Hepa1c1c7, Hepa1-6, LLC1 and A549 cells were obtained from ATCC. Hepa1c1c7 cells were cultured in Minimum Essential Media, Alpha Modification (Alpha MEM, #LM008-02, Welgene) supplemented with 10% fetal bovine serum (FBS, #16,000–044, Gibco), 100 units/mL penicillin, and 100 μg/mL streptomycin. Hepa1-6 and LLC1 cells were cultured in Dulbecco’s Modified Eagle Medium (DMEM, #LM001-05, Welgene) supplemented with 10% FBS, 100 units/mL penicillin, 100 μg/mL streptomycin. A549 cells were cultured in Rosewell Park Memorial Institute 1640 (RPMI 1640, #LM011-03, Welgene) supplemented with 10% FBS, 100 units/mL penicillin, and 100 μg/mL streptomycin. Cells were incubated at 37 °C under a 5% CO_2_-containing atmosphere.

### Cell viability assay

Cells were seeded at a density of 7 × 10^4^ cells/well in 96-well plates and incubated for 48 h. After washing with phosphate-buffered saline (PBS), cells were treated with various concentrations of compounds **1–5** or DMSO (vehicle control) and incubated for an additional 24 h. Subsequently, 4 µL of WST-1 solution was added to each well, followed by a 1 h incubation at 37 °C (Yeom et al. 2024; Zheng et al. 2024; Kim et al. 2025b). Absorbance at 450 nm was measured using a Tecan Spark microplate reader (Tecan Austria GmbH, Austria). Each condition was performed in triplicate.

### Western blot analysis

Cells were lysed using RIPA lysis buffer supplemented with phosphatase inhibitors. The lysates were resolved by SDS-PAGE and subsequently transferred to polyvinylidene fluoride (PVDF) membranes. Membranes were blocked with 2% skim milk in TBST at 4 °C and then incubated overnight at 4 °C with the following primary antibodies: MST1 (1:1000, #14,946, Cell Signaling Technology), SAV1 (1:1000, #13301S, Cell Signaling Technology), MOB1 (1:1000, #13730S, Cell Signaling Technology), LATS1 (1:1000, #3477S, Cell Signaling Technology), phospho-YAP (p-YAP; 1:1000, #4911S, Cell Signaling Technology), YAP (1:1000, #14,074, Cell Signaling Technology), TAZ (1:1000, #83669S, Cell Signaling Technology), pan-TEAD (1:1000, #13295S, Cell Signaling Technology), phospho-AKT (p-AKT; 1:1000, #9275S, Cell Signaling Technology), AKT (1:1000, #9272S, Cell Signaling Technology), phospho-ERK1/2 (p-ERK1/2; 1:1000, #PAB16949, Abnova), total ERK1/2 (1:1000, #9102S, Cell Signaling Technology), STAT3 (1:1000, #9139S, Cell Signaling Technology), Cyclin D1 (1:1000, sc-8396, Santa Cruz Biotechnology), Waf1/Cip1/CDKN1A p21 (1:1000, sc-53870, Santa Cruz Biotechnology), Bax (1:1000, sc-23959, Santa Cruz Biotechnology), Bcl-2 (1:1000, sc-23960, Santa Cruz Biotechnology), Caspase-3 (1:1000, sc-56053, Santa Cruz Biotechnology), and E-cadherin (1:1000, #3195, Cell Signaling Technology). After washing, membranes were incubated with HRP-conjugated secondary antibodies: anti-mouse IgG (1:2000, #7076P2, Cell Signaling Technology) or anti-rabbit IgG (1:2000, #7074S, Cell Signaling Technology). Proteins were visualized using an enhanced chemiluminescence (ECL) reagent (5,900,609, ATTO) and detected with the LuminoGraph II imaging system (WSE-6200, ATTO).

### Statistical analysis

All in vitro data were analyzed using two-tailed, unpaired *t* tests in GraphPad Prism 7 (GraphPad Software, La Jolla, CA, USA). Sample sizes for each group were n ≥ 3. Results are presented as mean ± SEM, with a significance threshold of *P* < 0.05. P values and detailed information for each experiment are provided in the figure legends.

## Results

### Isolation and structural identification of the compounds

**Fig. 1:**
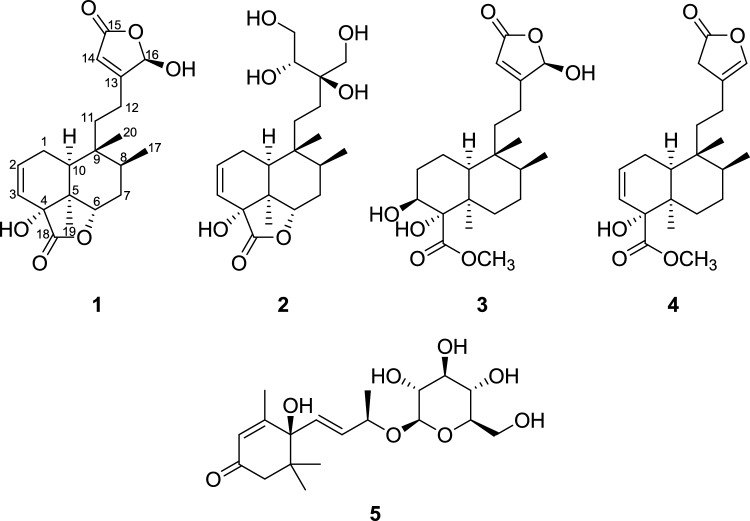
Chemical structure of the isolated compounds **1**–**5**

Leaves of *T. crispa* were extracted by Soxhlet with methanol (MeOH). The crude extract was subsequently partitioned using four solvents: *n*-hexane, dichloromethane (CH₂Cl₂), ethyl acetate, and *n*-butanol (*n*-BuOH). Among the resulting solvent-soluble fractions, the CH₂Cl₂- and *n*-BuOH-soluble fractions were further investigated using repeated column chromatography and high-performance liquid chromatography (HPLC), guided by LC/MS analysis combined with an in-house UV spectral library. Chemical investigation of these fractions led to the isolation of five compounds (**1**–**5**), including four new *cis*-clerodane-type diterpenoids (**1**–**4**) (Fig. 1).

**Fig. 2:**
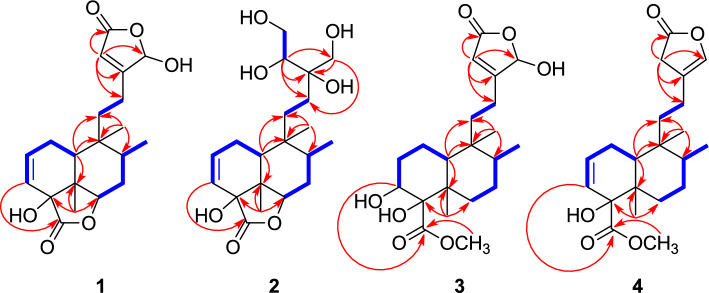
Key ^1^H-^1^H COSY (blue bold) and HMBC (red arrow) correlations for compounds **1**–**4**

Compound **1** was obtained as a colorless gum, and its molecular formula was determined to be C_20_H_26_O_6_ based on the [M − H]^−^ ion at *m/z* 361.1665 (calcd for C_20_H_25_O_6_, 361.1657) in negative-ion mode HR-ESIMS (Fig. S1). The ^1^H NMR data (Table 1) of **1**, assigned via HSQC, revealed characteristic signals, including three olefinic protons [*δ*_H_ 6.26 (1H, ddd, *J* = 9.5, 6.5, 3.0 Hz, H-2), 5.77 (1H, dd, *J* = 9.5, 3.0 Hz, H-3), and 5.93 (1H, s, H-14)], three methyl groups [*δ*_H_ 1.02 (3H, d, *J* = 7.0 Hz, H-17), 1.23 (3H, s, H-19), and 0.95 (3H, s, H-20)], and four methine groups [*δ*_H_ 4.58 (1H, dd, *J* = 12.0, 7.5 Hz, H-6), 2.20 (1H, m, H-8), 2.05 (1H, dd, *J* = 9.5, 2.5 Hz, H-10), and 6.06 (1H, br s, H-16)]. The ^13^C NMR data (Table 1) of **1**, supported by HMBC, revealed 20 carbon resonances, including four olefinic carbons [*δ*_C_ 135.5 (C-2), 126.8 (C-3), 172.3 (C-13), and 117.8 (C-14)], two ester carbonyl carbons [*δ*_C_ 173.6 (C-15) and 180.7 (C-18)], and three oxygen-attached carbons [*δ*_C_ 78.3 (C-4), 78.1 (C-6), and 101.0 (C-16)]. The ^1^H and ^13^C NMR data of **1** closely resembled those of the previously reported *cis*-clerodane-type diterpenoids tinotufolin D and tinopanoid M, with the decalin moiety similar to that of tinotufolin D and the *γ*-butyrolactone moiety corresponding to that of tinopanoid M (Zhu et al. 2023; Fukuda et al. 1994). The planar structure of **1** was elucidated from analysis of 2D NMR (^1^H–^1^H COSY and HMBC) data (Fig. 2). The *γ*-butyrolactone moiety was deduced from the key HMBC correlations of H-14 to C-13/C-15/C-16. In particular, the carbonyl carbon C-15 (*δ*_C_ 173.6) indicated the presence of an ester linkage. The chemical shifts of the olefinic proton and carbon, H-14 (*δ*_H_ 5.93) and C-14 (*δ*_C_ 117.8), suggested that the proton is positioned at the *α*-position in an α,β-unsaturated system. In addition, the downfield chemical shifts of H-16 (*δ*_H_ 6.06) and C-16 (*δ*_C_ 101.0) were attributed to the combined effects of the adjacent ester bond, olefinic bond, and a hydroxyl substituent. The point of attachment of the *γ*-butyrolactone moiety to the decalin core was confirmed by the key ^1^H–^1^H COSY correlations between H-11 and H-12, as well as HMBC correlations from H-14 to C-12. These findings indicate that the C-13 to C-16 region forms a *γ*-butyrolactone ring, a structural feature characteristic of clerodane-type diterpenoids commonly found in the genus *Tinospora* (Zhu et al. 2023). The decalin moiety was deduced from the key ^1^H–^1^H COSY correlations of H-10/H-1/H-2/H-3 and H-6/H-7/H-8/H-17 and HMBC correlations from H-10 to C-5/C-9/C-11, from H-19 to C-4/C-5/C-6, and from H-20 to C-8/C-9/C-11. Three methyl groups were determined to be attached to C-5, C-8, and C-9. A key HMBC correlation from H-3 to C-18 (*δ*_C_ 180.7) indicated the presence of an ester group at C-18, which is connected to C-4. This finding suggests that, unlike a typical clerodane diterpenoid bearing a methyl group at this position, C-18 in this compound is substituted with an ester moiety. The oxygenated carbon signal at C-6 (*δ*_C_ 78.1), which is more downfield than that of a typical hydroxyl-bearing carbon, suggests an ester linkage between C-4 and C-6 (Fukuda et al. 1994). These data collectively indicate that another *γ*-butyrolactone moiety is fused to the decalin core. Accordingly, the complete planar structure of compound **1** was determined.

**Fig. 3:**
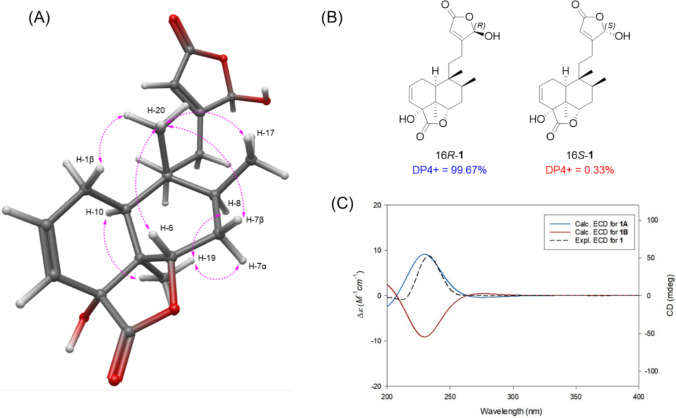
**A** Key NOESY correlations for **1**. **B** DP4 + analysis and probability scores for 16*R*-**1**/16*S*-**1**. **C** Experimental and calculated ECD spectra for **1**

The relative configuration of compound **1** was established through NOESY analysis (Fig. 3). NOESY correlations of H-19 with H-7α/H-8/H-10 and of H-20 with H-1β/H-6/H-7β/H-17 were observed. Notably, the NOESY correlation between H-19 and H-10 confirmed that compound **1** possesses a *cis*-clerodane skeleton. However, the configuration of hydroxyl group at C-4 could not be fully determined by NOESY alone. Therefore, the PANIC (peak amplitude normalization for improved cross-relaxation) method was employed. In the case of an α-oriented 4-OH, the calibrated interproton distance between H-6 and H-20 was measured to be 2.97 Å, which closely matched the predicted distance (3.25 Å) from the molecular-mechanics (MM)-optimized 3D structure, better than the β-oriented model (2.10 Å) (Fig. S9 and S10). To establish the absolute configuration at C-16, GIAO NMR chemical-shift calculations followed by the DP4 + probability approach were performed. The calculated ^1^H and ^13^C NMR chemical shifts for the two possible enantiomers, 16*R*-**1** and 16*S*-**1**, were compared with the experimental data, resulting in a 99.67% probability in favor of the 16*R*-**1** isomer (Fig. 3). Finally, to determine the absolute configuration of **1**, the ECD data of two possible isomers, **1A** (4*R*,5*S*,6*S*,8*S*,9*R*,10*S*,16*R*) and **1B** (4*S*,5*R*,6*R*,8*R*,9*S*,10*R*,16*S*), were calculated and compared with the experimental ECD data of compound **1** (Fig. 3). The experimental ECD data showed good agreement with the calculated spectrum of isomer **1A**, indicating that the absolute configuration of **1** is 4*R*,5*S*,6*S*,8*S*,9*R*,10*S*,16*R*. Accordingly, the chemical structure of compound **1**, including its absolute configuration, was fully elucidated and designated as tinospordane A.

**Fig. 4:**
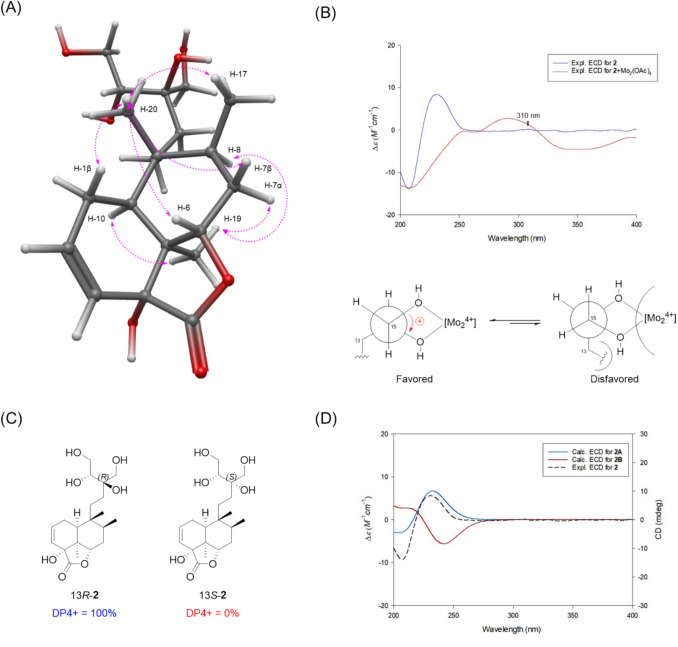
**A** Key NOESY correlations for** 2**. **B** Conformations of the Mo complex of **2**. The positive cotton effect of the complex at 310 nm confirmed the favored conformation as being the *R*-configuration at C-14. **C** DP4 + analysis and probability scores for 13*R*-**2**/13*S*-**2**. **D** Experimental and calculated ECD spectra for **2**

Compound **2** was determined to have the molecular formula C_20_H_32_O_7_ based on the [M − H]^−^ ion at *m/z* 383.2072 (calcd for C_20_H_31_O_7_, 383.2075) in negative-ion mode HR-ESIMS. The ^1^H and ^13^C NMR spectra (Table 1) of compound **2** closely resembled those of compound **1**, except for the side chain spanning C-11 to C-16. Unlike the *γ*-butyrolactone moiety observed in compound **1**, compound **2** lacks an ester bond, despite showing hydroxylated ^13^C chemical shifts for C-13 (*δ*_C_ 76.6), C-14 (*δ*_C_ 76.0), C-15 (*δ*_C_ 63.4), and C-16 (*δ*_C_ 64.5), as confirmed by HSQC and HMBC correlations. A key ^1^H–^1^H COSY correlation between H-14 and H-15, along with HMBC correlations from H-14 to C-12/C-13/C-16 and from H-16 to C-12/C-13, revealed that a tetritol moiety is connected to the decalin core at C-12 through C-13, rather than forming a *γ*-butyrolactone (Fig. 2). Accordingly, the complete planar structure of compound **2** was determined. The relative configuration of compound **2** was established by NOESY analysis (Fig. 4). As observed for compound **1**, NOESY correlations of H-19 with H-7α/H-8/H-10 and of H-20 with H-1β/H-6/H-7β/H-17 confirmed the *cis*-clerodane skeleton. To determine the configuration of hydroxyl group at C-4, the PANIC method was employed. In the case of an α-oriented 4-OH, the calibrated interproton distance between H-6 and H-20 was measured to be 3.02 Å, closely matching the predicted distance (3.24 Å) from the MM-optimized 3D structure and better than the β-oriented model (2.10 Å) (Fig. S19 and S20). The absolute configuration at C-14 was determined using Snatzke’s method (Di Bari et al. 2001; Jo et al. 2020). Upon formation of a Mo₂(OAc)₄ complex with the vicinal diol at C-15/C-14, a positive Cotton effect around 310 nm in the induced circular dichroism (ICD) spectrum of **2** was observed, indicating the *R* configuration at C-14 (Fig. 4). Subsequently, the configuration at C-13 was assigned via DP4 + probability analysis based on the calculated ^1^H and ^13^C NMR chemical shifts of two possible enantiomers (13*R*-**2** and 13*S*-**2**). The result supported 13*R*-**2** with a 100% probability (Fig. 4). Finally, the overall absolute configuration of compound **2** was established by comparing experimental ECD data with the calculated spectra of two candidate isomers: **2A** (4*R*,5*S*,6*S*,8*S*,9*R*,10*S*,13*R,*14*R*) and **2B** (4*S*,5*R*,6*R*,8*R*,9*S*,10*R*,13*S,*14*S*) (Fig. 4). The experimental ECD data showed good agreement with **2A**, confirming the absolute configuration of compound **2** as 4*R*,5*S*,6*S*,8*S*,9*R*,10*S*,13*R,*14*R*. Therefore, the complete structure, including stereochemistry, was fully elucidated, and the compound was designated as tinospordane B.

**Fig. 5:**
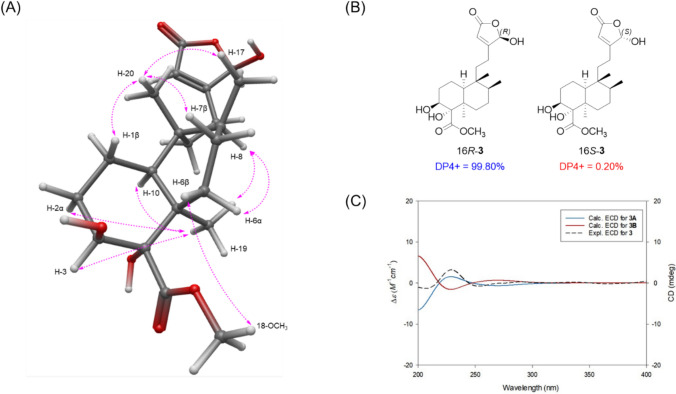
**A** Key NOESY correlations for **3**. **B** DP4 + analysis and probability scores for 16*R*-**3**/16*S*-**3**. **C** Experimental and calculated ECD spectra for **3**

Compound **3** was obtained as a colorless gum, and its molecular formula was determined to be C_21_H_32_O_7_ based on the [M − H]^−^ ion at *m/z* 395.2087 (calcd for C_21_H_31_O_7_, 395.2075) in negative-ion mode HR-ESIMS. The ^1^H and ^13^C NMR spectra (Table 1) of compound **3** closely resembled those of compound **1**, except for the decalin moiety spanning C-1 to C-10 and the presence of a methoxy group (18-OCH_3_), and also showed strong similarity to the previously reported tinotufolin C (Nishidono et al. 2024). Unlike compound **1**, compound **3** lacks the double bond between C-2 and C-3, which is replaced by a hydroxy group at C-3. This structural change was confirmed by key ^1^H–^1^H COSY correlations among H-10/H-1/H-2/H-3. In addition, the ester linkage via C-18 that connects C-4 and C-6 in compound **1** is absent in compound **3**, where a methoxy group is attached to C-18 instead. This was supported by key HMBC correlations of 18-OCH_3_/C-18 and H-3/C-18, and by ^1^H–^1^H COSY correlations among H-6/H-7/H-8/H-17. Accordingly, the complete planar structure of compound **3** was determined. The relative configuration of compound **3** was established by NOESY analysis (Fig. 5). NOESY correlations of H-19 with H-2α/H-3/H-8/H-10, of H-8 with H-6α, and of H-20 with H-1β/H-7β/H-17 confirmed the *cis*-clerodane skeleton. In addition, the NOESY correlation of 18-OCH₃ with H-6β indicated that the methyl ester group attached to C-4 is β-oriented. The configuration at C-16 was determined by DP4 + probability analysis using the calculated ^1^H and ^13^C NMR chemical shifts of two possible enantiomers (16*R*-**3** and 16*S*-**3**). The result supported 16*R*-**3** with a 99.80% probability (Fig. 5). The overall absolute configuration of compound **3** was further confirmed by comparison of the experimental and calculated ECD spectra for two enantiomers, **3A** (3*S*,4*S*,5*R*,8*S*,9*R*,10*S*,16*R*) and **3B** (3*R*,4*R*,5*S*,8*R*,9*S*,10*R*,16*S*) (Fig. 5). The experimental ECD data showed good agreement with **3A**, confirming the absolute configuration of compound **3** as 3*S*,4*S*,5*R*,8*S*,9*R*,10*S*,16*R*. Therefore, the complete structure, including stereochemistry, was fully elucidated, and the compound was designated as tinospordane C.

**Fig. 6:**
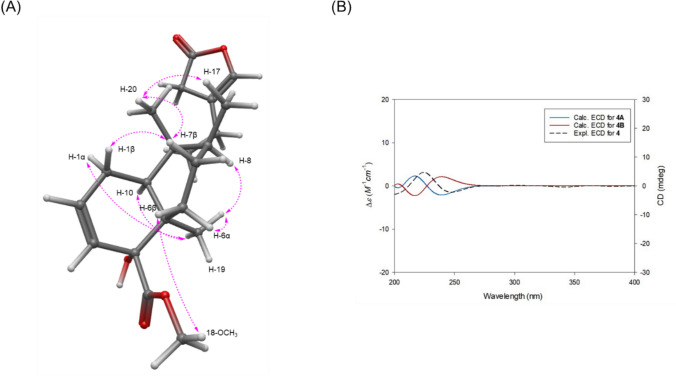
**A** Key NOESY correlations for **4**. **B** Experimental and calculated ECD spectra for **4**

Compound **4** was obtained as a colorless gum, and its molecular formula was determined to be C_21_H_30_O_5_ based on the [M + H]⁺ ion at *m/z* 363.2166 (calcd for C_21_H_31_O_5_, 363.2166) in positive-ion mode HR-ESIMS. The ^1^H and ^13^C NMR spectra (Table 1) of compound **4** closely resembled those of compound **3**, except for the *γ*-butyrolactone spanning C-13 to C-16 and the presence of a double bond between C-2 and C-3. In compound **4**, a key HMBC correlation from H-14 to C-12/C-13/C-15/C-16 confirmed the presence of the *γ*-butyrolactone moiety, similar to compound **3**. However, unlike compound **3**, a double bond was located between C-13 and C-16, as evidenced by the downfield chemical shifts of the oxygenated olefinic carbon (*δ*_C_ 139.6) and proton (*δ*_H_ 6.89) at C-16. In addition, the upfield chemical shifts of the carbon (*δ*_C_ 47.8) and proton (*δ*_H_ 3.92) at C-14, compared to those of compound **3**, indicated hydrogenation at C-14. Additionally, weak long-range couplings among H-14, H-16, and H-12 were observed in the ^1^H–^1^H COSY spectrum. The presence of a double bond between C-2 and C-3 was confirmed by sequential ^1^H–^1^H COSY correlations from H-10/H-1/H-2/H-3 (Fig. 2), consistent with the patterns observed in compounds **1** and **2**. Accordingly, the complete planar structure of compound **4** was determined. The relative configuration of compound **4** was established by NOESY analysis (Fig. 6). NOESY correlations of H-19 with H-1α/H-6α/H-8/H-10, of H-20 with H-7β/H-17, and of H-7β with H-1β confirmed the *cis*-clerodane skeleton. In addition, the NOESY correlation of 18-OCH₃ with H-6β indicated that the methyl ester group attached to C-4 is β-oriented. The absolute configuration of compound **4** was further confirmed by comparison of the experimental and calculated ECD spectra for two enantiomers, **4A** (4*R*,5*R*,8*S*,9*R*,10*S*) and **4B** (4*S*,5*S*,8*R*,9*S*,10*R*) (Fig. 6). The experimental ECD data showed good agreement with **4A**, confirming the absolute configuration of compound **4** as 4*R*,5*R*,8*S*,9*R*,10*S*. Therefore, the complete structure, including stereochemistry, was fully elucidated, and the compound was designated as tinospordane D.

The other isolated compound was identified as a megastigmane glycoside, (6*S*,9*S*)-roseoside (**5**) (Yajima et al. 2009), by comparison of its NMR spectra with reported data and by LC/MS analysis (Fig. S38 and S39).

### Anticancer efficacy of the isolated compounds 1–5 in liver and lung cancer cell lines

**Fig. 7:**
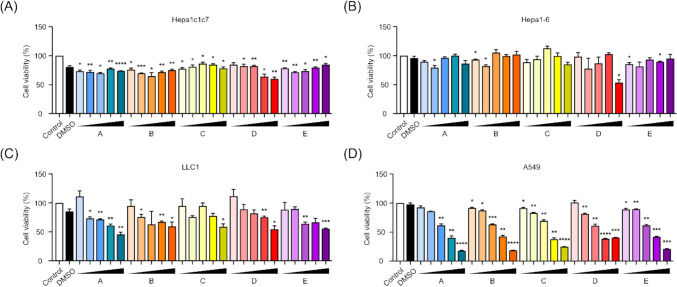
Dose-dependent effects of compounds **1**–**5** on cancer cell viability. WST-1 cell viability assay was performed to evaluate the cytotoxic effect of five compounds **1**–**5** (A = **5**, B = **2**, C = **1**, D = **3**, E = **4**) in liver and lung cancer cell lines following 24 h treatment at increasing concentrations (0, 10, 50, 100, 150, and 200 µM). **A** Hepa1c1c7 and **B** Hepa1-6 liver cancer cells exhibited only modest reductions in viability, even at the highest concentration, with no clear dose-dependent response. In contrast, a marked and dose-dependent decrease in viability was observed in lung cancer cells, including **C** LLC1 and **D** A549, particularly upon treatment with compounds **1**–**5** (A = **5**, B = **2**, C = **1**, D = **3**, E = **4**) showed inhibitory effects, though slightly less pronounced. Data represent mean ± SEM from three independent experiments. Statistical analysis was performed using a two-tailed unpaired t-test. Data are presented as mean ± SEM. *P < 0.05, **P < 0.01, ***P < 0.001, ****P < 0.0001

To evaluate the anticancer potential, the isolated compounds from *T. crispa* leaves were assessed in both liver (Hepa1c1c7, Hepa1-6) and lung (LLC1, A549) cancer cell lines using the WST-1 cell viability assay (Lee et al. 2025). Cells were treated with a range of concentrations (10–200 μM), and viability was measured after 24 h. In liver cancer cells (Hepa1c1c7 and Hepa1-6), all five compounds exhibited only mild cytotoxic effects across the tested concentration range (Fig. 7). Even at 200 μM, the maximum tested concentration, cell viability remained relatively high (> 70% of control), indicating limited sensitivity of these liver cancer cell lines to the compounds. Moreover, no clear dose-dependent effect was observed, suggesting low therapeutic efficacy of these compounds in hepatic cancer models under these conditions. In contrast, treatment with the compounds led to a marked reduction in cell viability in lung cancer cell lines (LLC1 and A549), particularly at higher concentrations. At 200 μM, A549 cells treated with compounds **1**, **2**, **4**, and **5** exhibited approximately 70% reduction in viability compared to untreated controls, while compound **3** also exerted inhibitory effects, albeit to a slightly lesser extent (Fig. 7D). Similar trends were observed in LLC1 cells (Fig. 7C). Notably, the reduction in viability was concentration dependent, highlighting a selective and dose-responsive anticancer effect of the compounds in lung cancer cells. Collectively, these findings indicate that isolated compounds exhibit selective cytotoxicity toward lung cancer cell lines, with significantly greater potency compared to liver cancer models, and that the inhibitory effect is positively correlated with compound concentration.

### Activation of Hippo signaling in liver cancer and suppression of prosurvival pathways in lung cancer cells

**Fig. 8:**
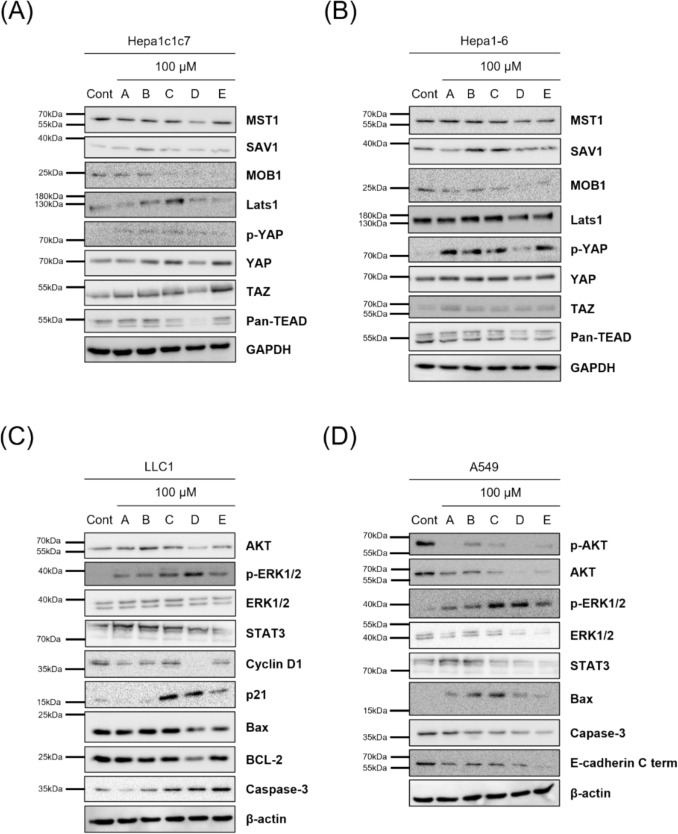
Effects of compounds **1**–**5** on cancer signaling pathways in liver and lung cancer cell lines. **A** Hepa1c1c7, **B** Hepa1-6, **C** LLC1, and **D** A549 cells were treated with 100 μM of compounds **1**–**5** (A = **5**, B = **2**, C = **1**, D = **3**, E = **4**) for 24 h. In liver cancer models, compounds **1** and **2** increased SAV1 and p-LATS1, while compound **3 **reduced YAP, TAZ, and pan-TEAD expression. Compounds **1**, **2**, **4**, and **5** elevated p-YAP. In lung cancer models, all compounds suppressed p-AKT; compounds **3** and **4** most reduced p-AKT, STAT3, Cyclin D1 and increased p21

To assess the molecular effects of compounds isolated from *T. crispa*, liver (Hepa1c1c7, Hepa1-6) and lung (LLC1, A549) cancer cell lines were treated with each compound at 100 μM for 24 h, and the expression and phosphorylation status of key cancer-related signaling components were evaluated by Western blot (Fig. 8A, D).

Treatment with compounds appeared to modulate Hippo signaling in liver cancer cells (Fig. 8A, B). While MST1 and MOB1 protein levels remained unchanged, compounds **1** and **2** increased SAV1 and p-LATS1, consistent with partial activation of the upstream kinase cascade. In contrast, compound **3** affected downstream signaling by reducing total YAP, TAZ, and pan-TEAD expression, while compounds **1**, **2**, **4**, and **5** elevated p-YAP levels. These results suggest that compounds **1** and **2** primarily enhance upstream Hippo activity, whereas compound **3** may suppress downstream transcriptional activity potentially through changes in YAP/TAZ levels and reduced TEAD-associated signaling. The differential modulation of upstream and downstream Hippo components suggests that both canonical kinase activity and post-translational processes, such as ubiquitin-mediated degradation, may be involved.

In lung cancer cells, the compounds broadly inhibited pro-survival signaling and promoted apoptotic pathways (Fig. 8C, D). All tested compounds suppressed phosphorylated AKT (p-AKT) without altering total AKT, with compounds **3** and **4** showing the strongest reductions. Although p-ERK1/2 levels were not substantially affected, total ERK1/2 expression declined in response to compounds **3**, **4**, and **5**. Compounds **1**, **3**, and **4** also downregulated STAT3 and Cyclin D1, accompanied by upregulation of the CDK inhibitor p21, consistent with induction of G1 cell cycle arrest. Furthermore, Bax and cleaved caspase-3 were elevated, whereas anti-apoptotic BCL-2 was markedly reduced, particularly by compound **3**. In addition, compounds **3** and **4** increased E-cadherin expression, indicative of suppression of epithelial-to-mesenchymal transition (EMT). Together, these data suggest that the compounds reduce oncogenic signaling while promoting apoptosis and epithelial characteristics in lung cancer cells. Band intensities from Western blot analyses were quantified across three independent biological replicates (*n* = 3), and the corresponding bar graphs are provided in the Supplementary Data (Fig. S40).

## Discussion

The present study provides new insights into the anticancer potential of *T. crispa*, a medicinal plant traditionally used in Southeast Asia. Through comprehensive phytochemical investigation, four new *cis*-clerodane-type diterpenoids (tinospordanes A–D) were isolated alongside one known compound. Among these, compounds **1**, **3**, and **4** exhibited notable anticancer effects across both liver and lung cancer models, indicating that *cis*-clerodane-type diterpenoids represent a structurally unique class of bioactive molecules with promising lead candidates.

Compound **3** emerged as the most potent and consistent candidate, displaying a cell-line specific anticancer effect. In hepatocellular carcinoma cells, it robustly activated the Hippo signaling pathway, a critical regulator of cell proliferation and tumor suppression. Activation of this pathway suggests that compound **3** may restore growth control in liver cancer cells by enhancing YAP/TAZ regulation, a process often dysregulated in hepatocellular carcinoma. In lung cancer models, compound **3** simultaneously suppressed multiple pro-survival signaling pathways, including AKT, ERK, STAT3, and BCL-2, leading to apoptotic cell death. This multi-targeted action highlights its therapeutic versatility, addressing both uncontrolled proliferation and resistance mechanisms commonly encountered in cancer treatment. Such cell line–specific anticancer effect underscores the potential utility of compound **3** as a preliminary lead rather than a fully optimized therapeutic candidate, particularly given the relatively high concentrations (~ 200 µM) required to elicit biological responses. Such cell-line specific anticancer effect underscores the potential of compound **3** as a lead scaffold for the development of novel anticancer agents with broader applicability.

These findings also expand the pharmacological relevance of *T. crispa*, which has traditionally been associated with immunomodulatory and anti-inflammatory activities. The discovery of potent anticancer *cis*-clerodanes adds a new dimension to its therapeutic profile, aligning with growing interest in natural products as sources of structurally diverse anticancer agents. The distinct biological profiles of compounds **1**–**4** highlight structure–activity relationships (SAR) that govern their modulation of oncogenic signaling pathways. Compounds **1** and **2**, which preserve the C-4 to C-6 ester linkage via C-18, predominantly influence the Hippo pathway at an upstream level, suggesting that this structural feature favors regulatory control through SAV1-mediated LATS1 activation. In contrast, hydrolysis of this ester linkage, as observed in compound **3**, appears to shift the mode of action toward direct downstream interference, facilitating effective suppression of YAP/TAZ–TEAD signaling. Notably, the diminished activity of compound **4**, despite sharing the cleaved ester linkage with compound **3**, indicates that ester hydrolysis alone does not fully account for potent Hippo pathway inhibition. Additional structural elements, particularly the maintenance of hydroxyl substitutions, seem to play a crucial role in stabilizing downstream target engagement and maximizing pathway suppression. These observations collectively suggest that cooperative contributions from both ester linkage status and hydroxyl group configuration are required for optimal activity. Beyond Hippo signaling, compounds bearing the hydrolyzed ester motif exert broader regulatory effects on multiple pro-survival pathways, consistent with a multi-target mechanism that converges on cell cycle control and apoptotic regulation. Taken together, these findings establish ester linkage hydrolysis as a pivotal structural switch that enables expanded signaling interference, while highlighting the importance of auxiliary functional groups in fine-tuning the overall anticancer efficacy of *cis*-clerodane diterpenoids. Subtle modifications to the *cis*-clerodane scaffold may enhance potency, selectivity, and pharmacokinetic properties, paving the way for drug development.

In conclusion, this study highlights the potential of *cis*-clerodane-type diterpenoids from *T. crispa*, particularly compound **3**, as promising leads for anticancer drug discovery. The multitargeting properties of compound **3**—activating tumor-suppressive Hippo signaling in liver cancer while inhibiting pro-survival pathways in lung cancer—offers a compelling mechanistic framework for future research. Moving forward, in vivo efficacy studies, comprehensive toxicity assessments, and mechanistic elucidation will be critical to translating these findings into clinically relevant anticancer therapeutics.

## Supplementary Information

Below is the link to the electronic supplementary material.

Supplementary file1 (DOCX 4162 KB)

## Data Availability

The data are available upon reasonable request from the author.
