# Supplementary material for: cis-Clerodane-type diterpenoids from Tinospora crispa and their anticancer potential
Source: Arch Pharm Res. 2026 Jan 20;49(1):158–73. doi: 10.1007/s12272-026-01596-y (PMC12913334; doi:10.1007/s12272-026-01596-y)
Supplement: Supplementary file 1 — Supplementary file1 (DOCX 4162 KB) [file 12272_2026_1596_MOESM1_ESM.docx]

| **Supporting Information** |
| --- |

***cis*-Clerodane-type diterpenoids from *Tinospora crispa*** **and their anticancer potential**

Se Yun Jeong ^1,a^, Jisun Kim ^2,a^, Ji Won Ha ^1^, Norhayati Ahmad ^3^, Nurul Hazlina Zaini ^4^, Yoon-Joo Ko ^5^, Alan Jung Park ^6^, Wonhwa Lee ^2,^* and Ki Hyun Kim ^1,^*

^1^ School of Pharmacy, Sungkyunkwan University, Suwon 16419, Republic of Korea

^2^ Department of Chemistry, Sungkyunkwan University, Suwon, 16419, Republic of Korea

^3^ Institute for Biodiversity and Environmental Research, Universiti Brunei Darussalam, Jalan Tunku Link Gadong, BE1410, Brunei Darussalam

^4^ Universiti Brunei Darussalam Botanical Research Centre, Institute for Biodiversity and Environmental Research, Universiti Brunei Darussalam, Jalan Tunku Link, Gadong BE1410, Brunei Darussalam

^5^ Laboratory of Nuclear Magnetic Resonance, National Center for Inter-University Research Facilities (NCIRF), Seoul National University, Seoul 08826, Republic of Korea

^6^ Department of Physiology, Seoul National University College of Medicine, Seoul, Republic of Korea

^a^ These authors contributed equally to this study.

*** Corresponding author: Ki Hyun Kim, Tel: +82-31-290-7700; Fax: +82-31-290-7730; E-mail: khkim83@skku.edu; Wonhwa Lee, E-mail: wonhwalee@skku.edu

**Supporting Information Contents:**

**Figure S1.** The HR-ESIMS data of **1**……………………………………………………………………………………………………………S4

**Figure S2.** The UV spectrum of **1**……………………………………………………………………………………………………………… S5

**Figure S3.** The ^1^H NMR spectrum of **1** (CD_3_OD, 850 MHz) ………………………………...…………………………………………………S6

**Figure S4.** The ^13^C NMR spectrum of **1** (CD_3_OD, 212.5 MHz) …………………………………………………………………………………S7

**Figure S5.** The ^1^H-^1^H COSY spectrum of **1**……………………………………………………………………………………………………S8

**Figure S6**. The HSQC spectrum of **1**…………………………………………………….…………………………………………………….…S9

**Figure S7.** The HMBC spectrum of **1**………………………….………………………………………………………………………………S10

**Figure S8.** The NOESY spectrum of **1**………………………….………………………………………………………………………………S11 **Figure S9.** 2D NOESY spectrum slice showing irradiation at H-6 in **1** …………………………………………………………………………S12

**Figure S10.** Calculated Interproton distance between H-6 and H-20 based on the different possible orientation of the 4-OH in compound **1**S13

**Figure S11.** The DP4+ analysis for **1**………………………….………………………………………………………………………………S14

**Figure S12.** The HR-ESIMS data of **2**…………………………………………………………………………………………………………S15

**Figure S13.** The UV spectrum of **2**……………………………………………………………………………………………………………S16

**Figure S14.** The ^1^H NMR spectrum of **2** (CD_3_OD, 850 MHz) ………………………………...………………………………………………S17

**Figure S15.** The ^1^H-^1^H COSY spectrum of **2**…………………………………………………………………………………………………S18

**Figure S16**. The HSQC spectrum of **2**………………………………………………….…………………………………………………….…S19

**Figure S17.** The HMBC spectrum of **2**………………………….………………………………………………………………………………S20

**Figure S18.** The NOESY spectrum of **2**………………………….……………………………………………………………………………S21

**Figure S19.** 2D NOESY spectrum slice showing irradiation at H-6 in **2** ………………………………………………………………………S22

**Figure S20.** Calculated Interproton distance between H-6 and H-20 based on the different possible orientation of the 4-OH in compound **2**S23

**Figure S21.** The DP4+ analysis for **2**………………………….………………………………………………………………………………S24

**Figure S22.** The HR-ESIMS data of **3**…………………………………………………………………………………………………………S25

**Figure S23.** The UV spectrum of **3**…………………………………………………………………………………………………………… S26

**Figure S24.** The ^1^H NMR spectrum of **3** (CD_3_OD, 850 MHz) ……………………………...…………………………………………………S27

**Figure S25.** The ^1^H-^1^H COSY spectrum of **3**…………………………………………………………………………………………………S28

**Figure S26**. The HSQC spectrum of **3**……………………………………………….……………………………………………………….…S29

**Figure S27.** The HMBC spectrum of **3**……………………….…………………………………………………………………………………S30

**Figure S28.** The NOESY spectrum of **3**……………………….………………………………………………………………………………S31

**Figure S29.** The DP4+ analysis for **3**………………………….………………………………………………………………………………S32

**Figure S30.** The HR-ESIMS data of **4**…………………………………………………………………………………………………………S33

**Figure S31.** The UV spectrum of **4**…………………………………………………………………………………………………………… S34

**Figure S32.** The ^1^H NMR spectrum of **4** (CD_3_OD, 850 MHz) ……………………………...…………………………………………………S35 **Figure S33.** The ^13^C NMR spectrum of **4** (CD_3_OD, 212.5 MHz) ………………………………………………………………………………S36

**Figure S34.** The ^1^H-^1^H COSY spectrum of **4**…………………………………………………………………………………………………S37

**Figure S35.** The HSQC spectrum of **4**……………………………………………….……………………………………………………….…S38

**Figure S36.** The HMBC spectrum of **4**……………………….…………………………………………………………………………………S39

**Figure S37.** The NOESY spectrum of **4**……………………….………………………………………………………………………………S40

**Figure S38.** The negative ion mode LC/MS data of **5**…………………………………………………………………………………………S41

**Figure S39.** The ^1^H NMR spectrum of **5** (CD_3_OD, 850 MHz) …………………………………………………………………………………S42

**Figure S40.** Quantification of Western blot band intensities … …………………………………………………………………………….…S43

**Figure S1.** The HR-ESIMS data of **1**

**
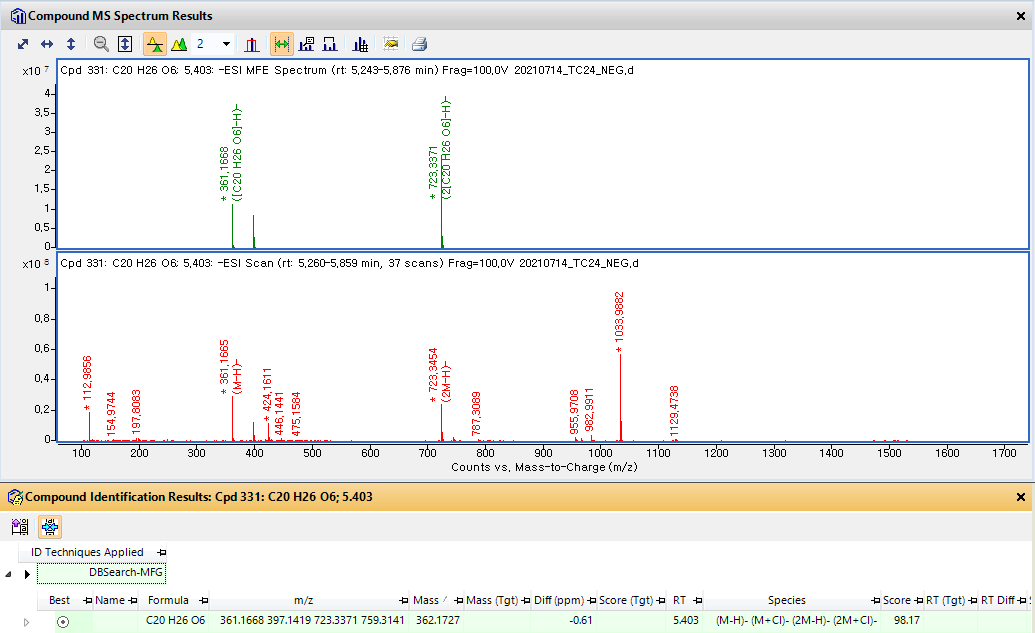
**

**Figure S2.** The UV spectrum of **1**


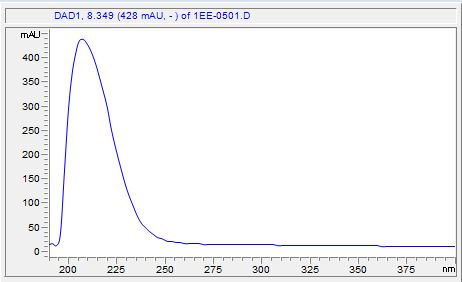


**Figure S3.** The ^1^H NMR spectrum of **1** (CD_3_OD, 850 MHz)


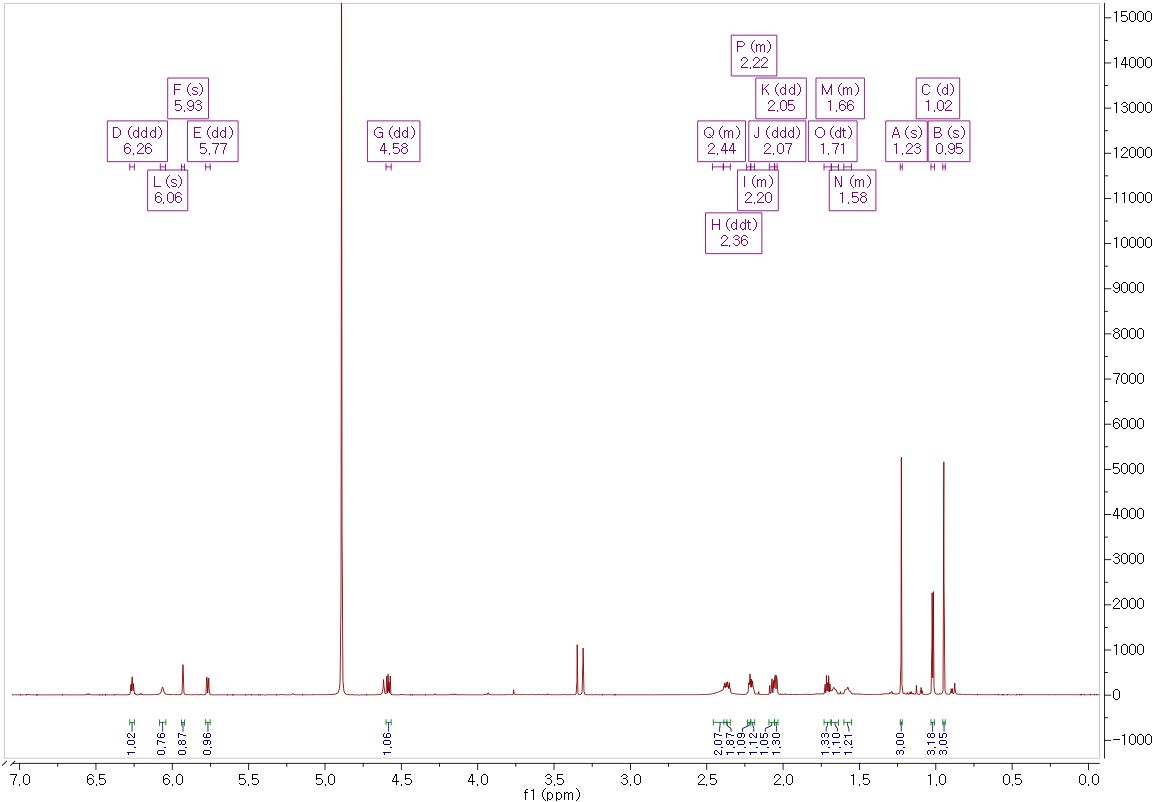


**Figure S4.** The ^13^C NMR spectrum of **1** (CD_3_OD, 212.5 MHz)


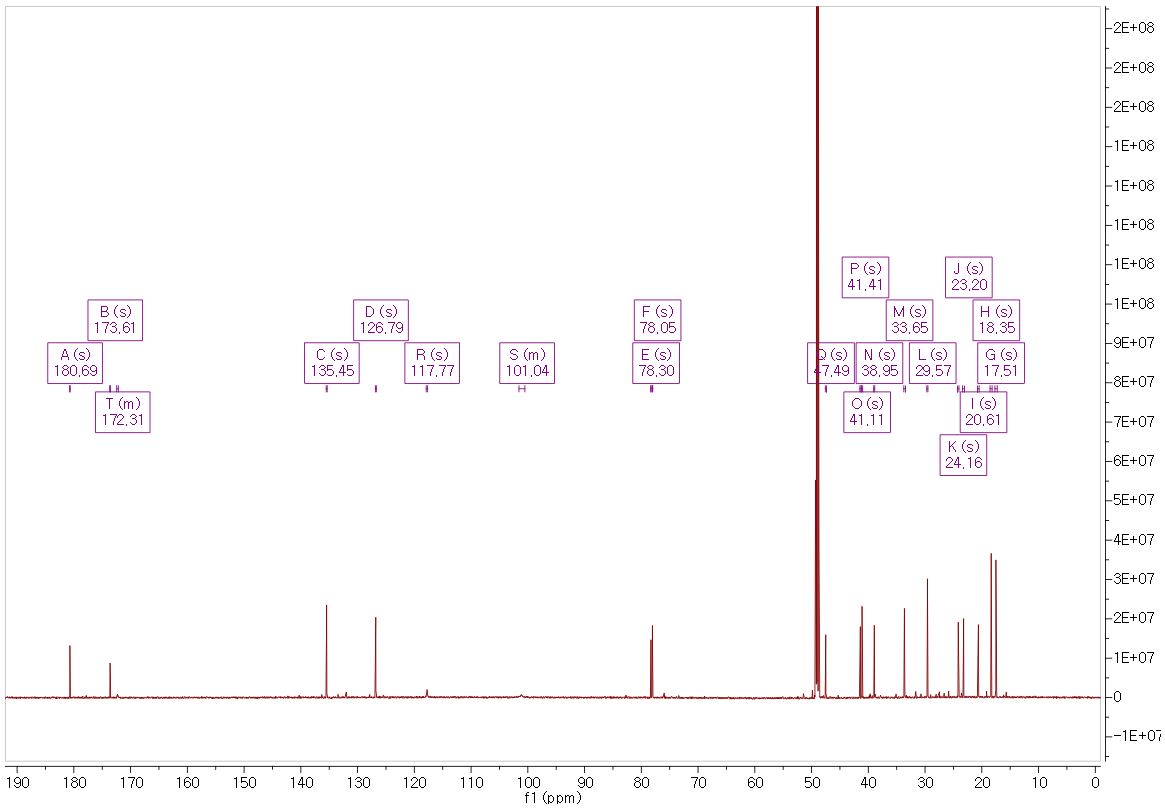


**Figure S5.** The ^1^H-^1^H COSY spectrum of **1**


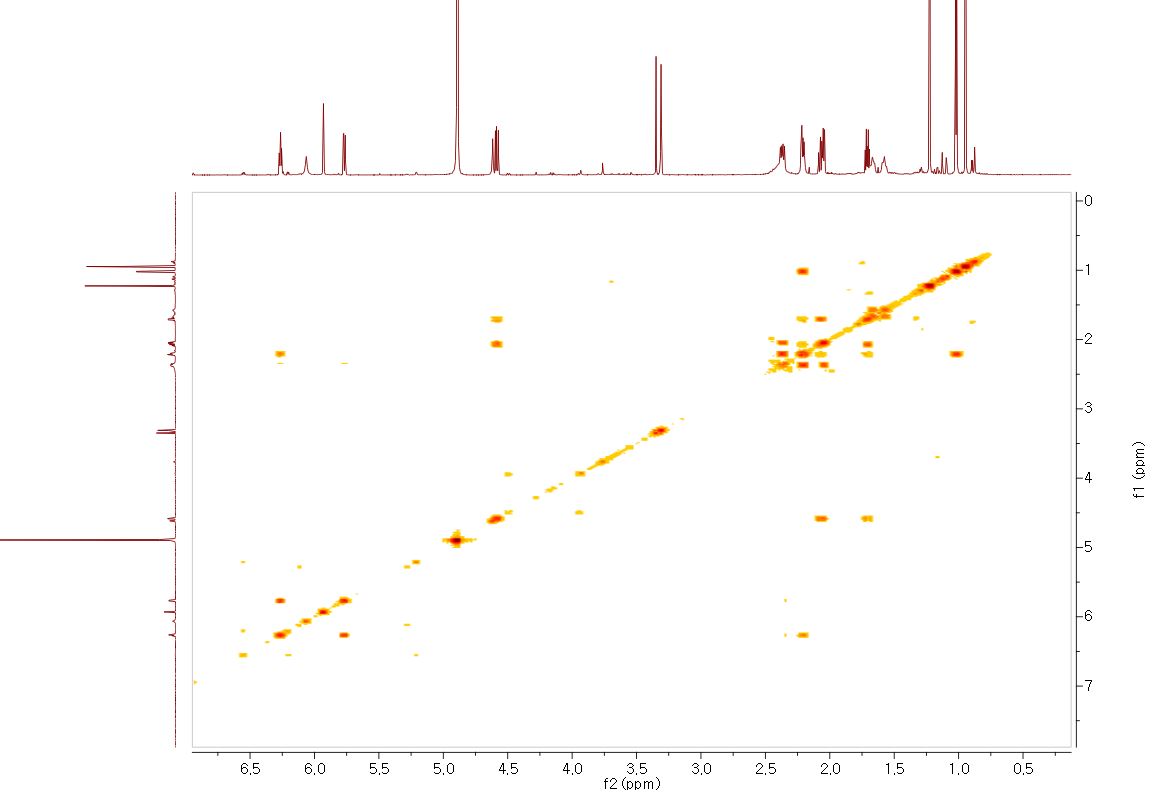


**Figure S6.** The HSQC spectrum of **1**

**
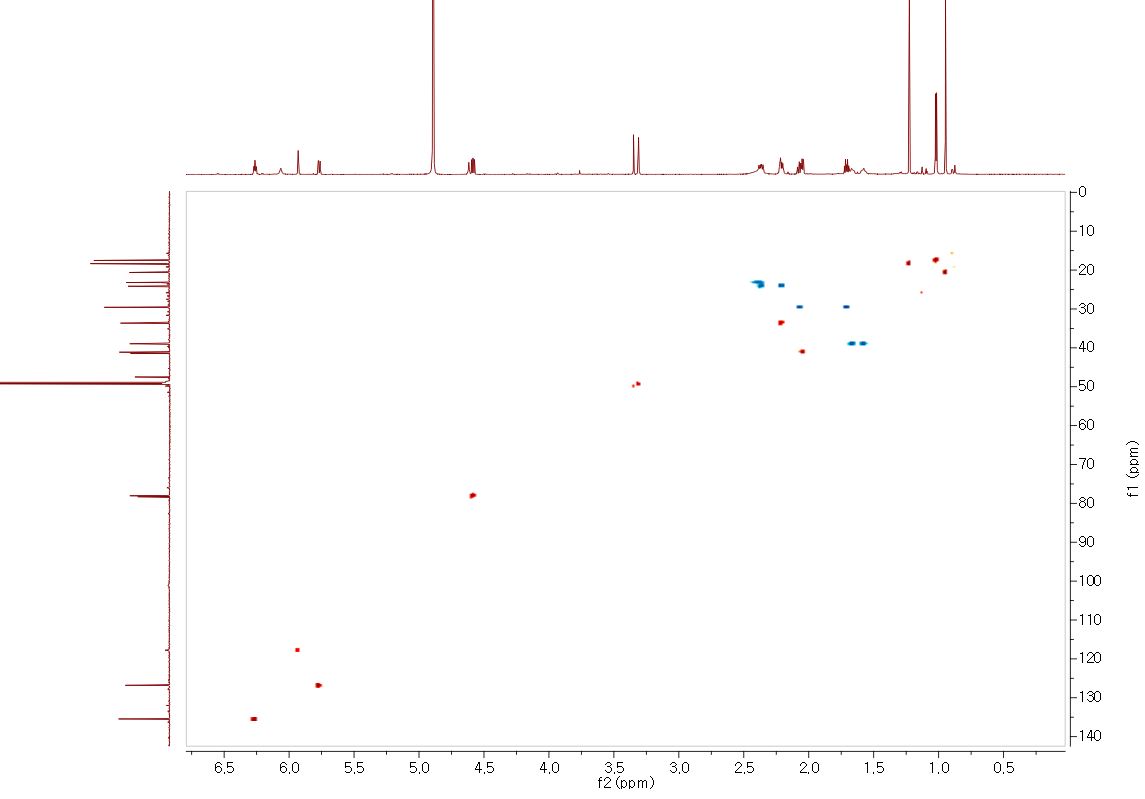
**

**Figure S7.** The HMBC spectrum of **1**

**
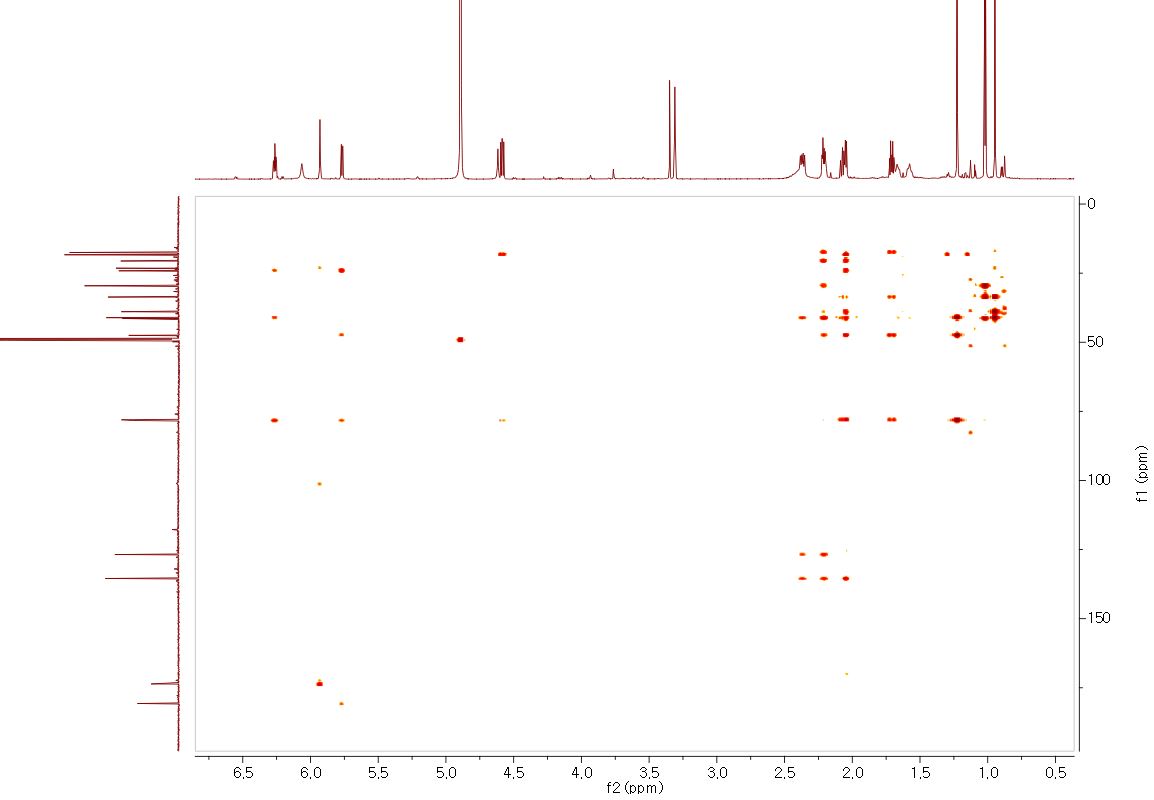
**

**Figure S8.** The NOESY spectrum of **1**


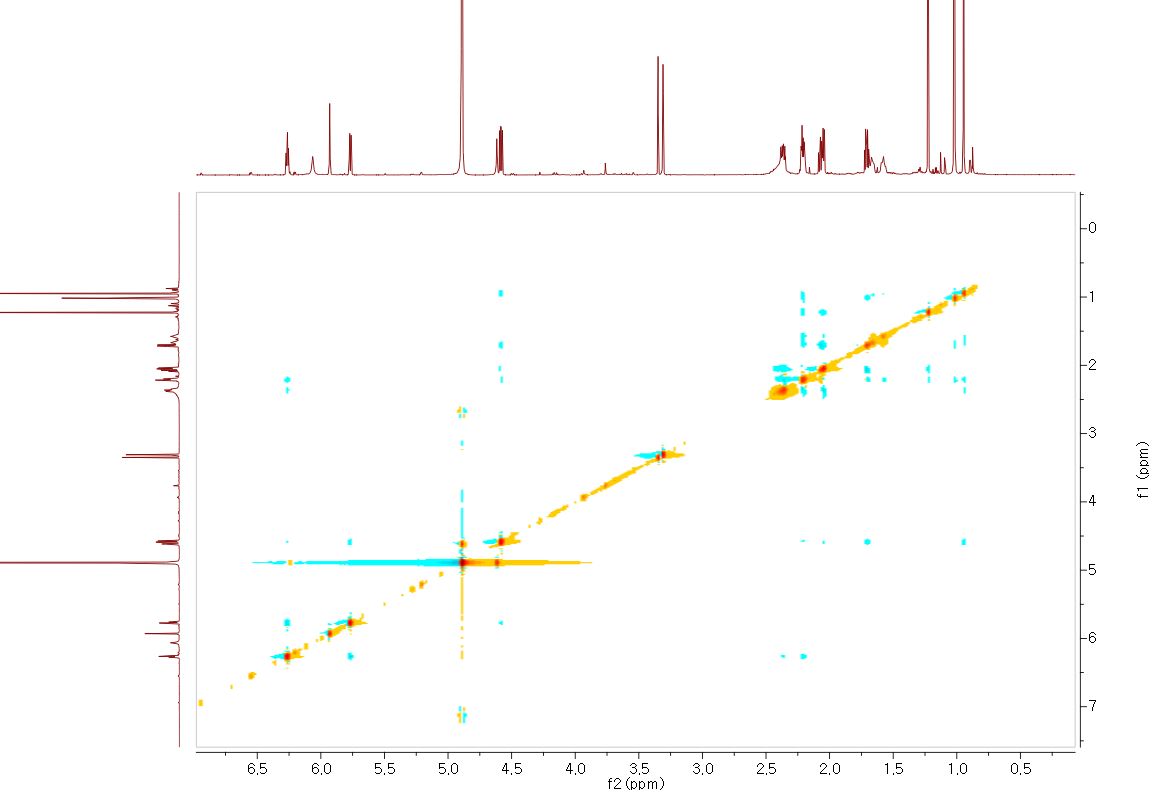


**Figure S9.** 2D NOESY spectrum slice showing irradiation at H-6 in **1**

**
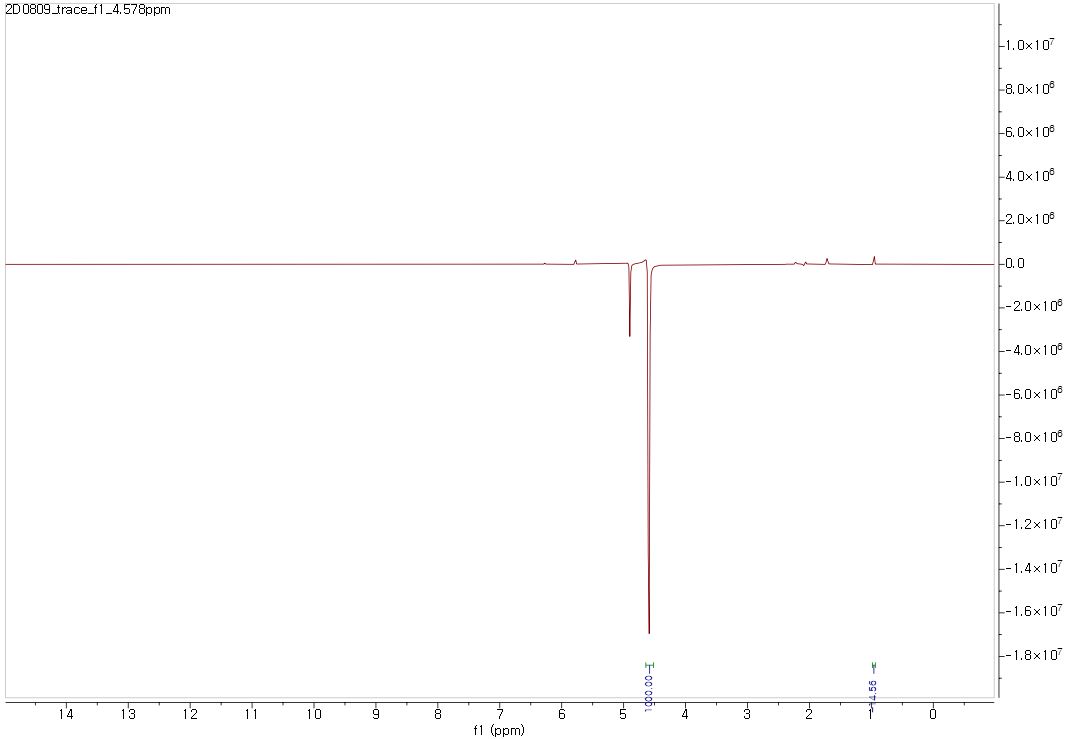
**

H-20

14.56

**Figure S10.** Calculated Interproton distance between H-6 and H-20 based on the different possible orientation of the 4-OH in compound **1**

**
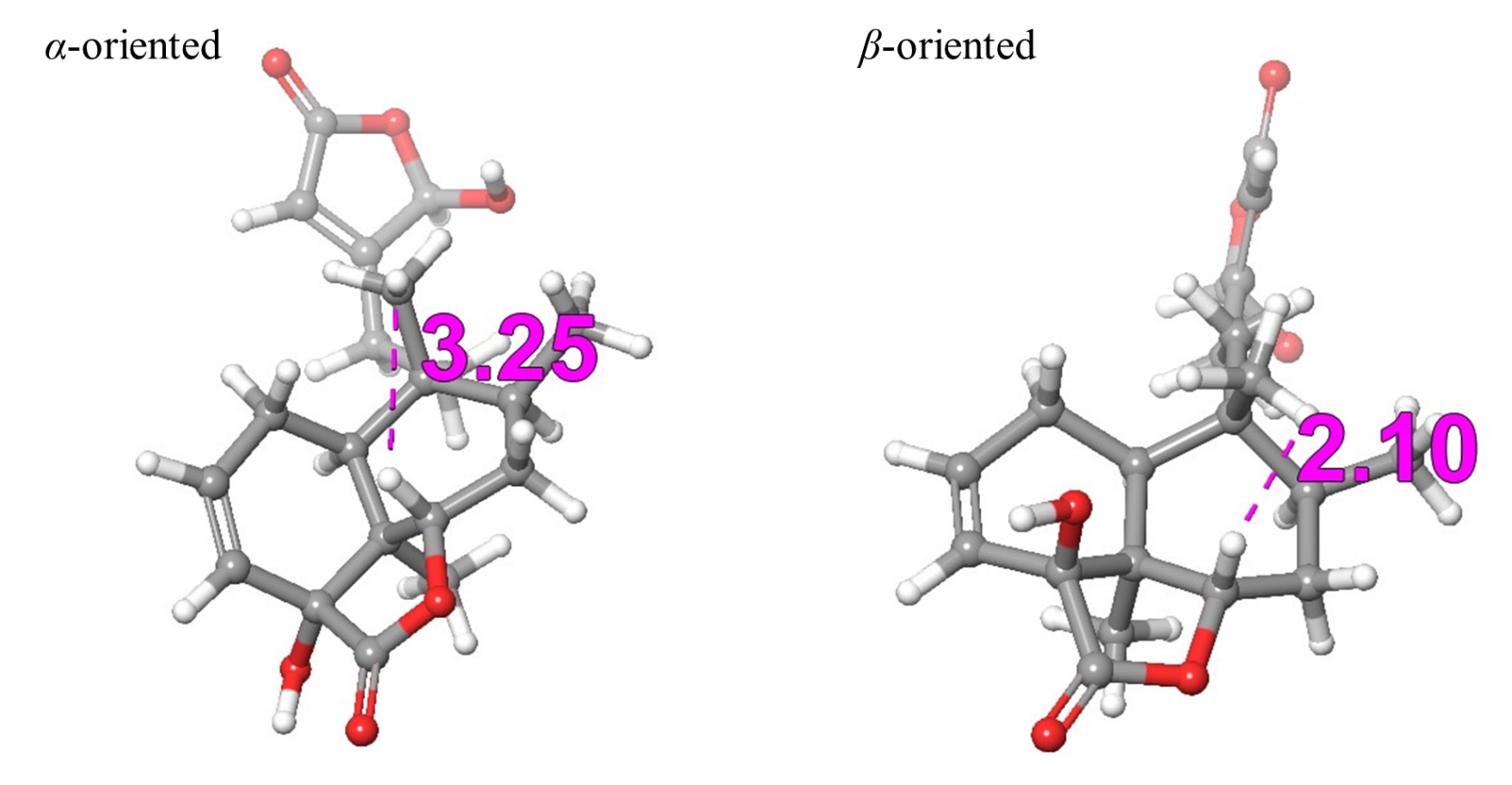
**

**Figure S11.** The DP4+ analysis for **1**


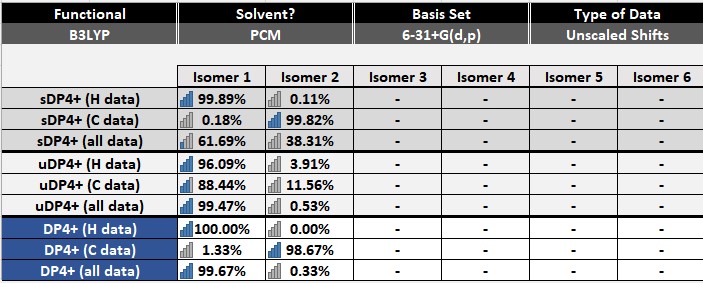


**Figure S12.** The HR-ESIMS data of **2**

**
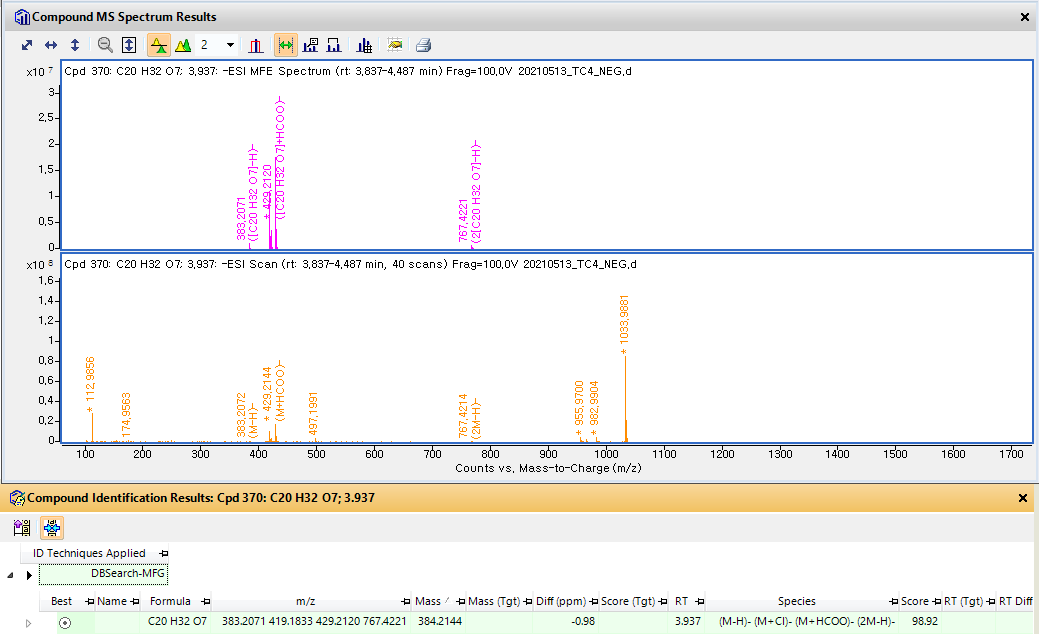
**

**Figure S13.** The UV spectrum of **2**


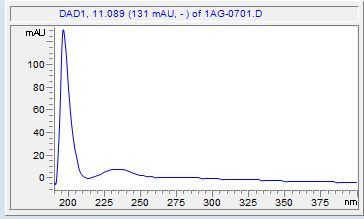


**Figure S14.** The ^1^H NMR spectrum of **2** (CD_3_OD, 850 MHz)


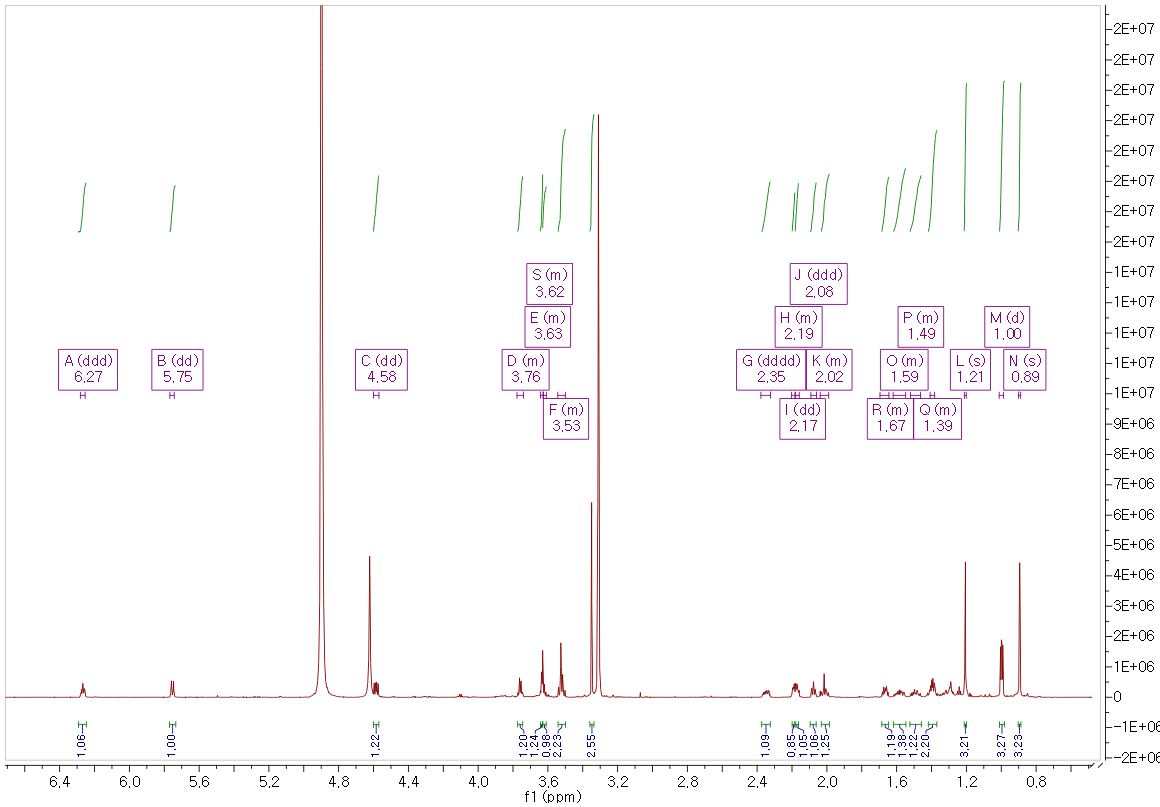


**Figure S15.** The ^1^H-^1^H COSY spectrum of **2**


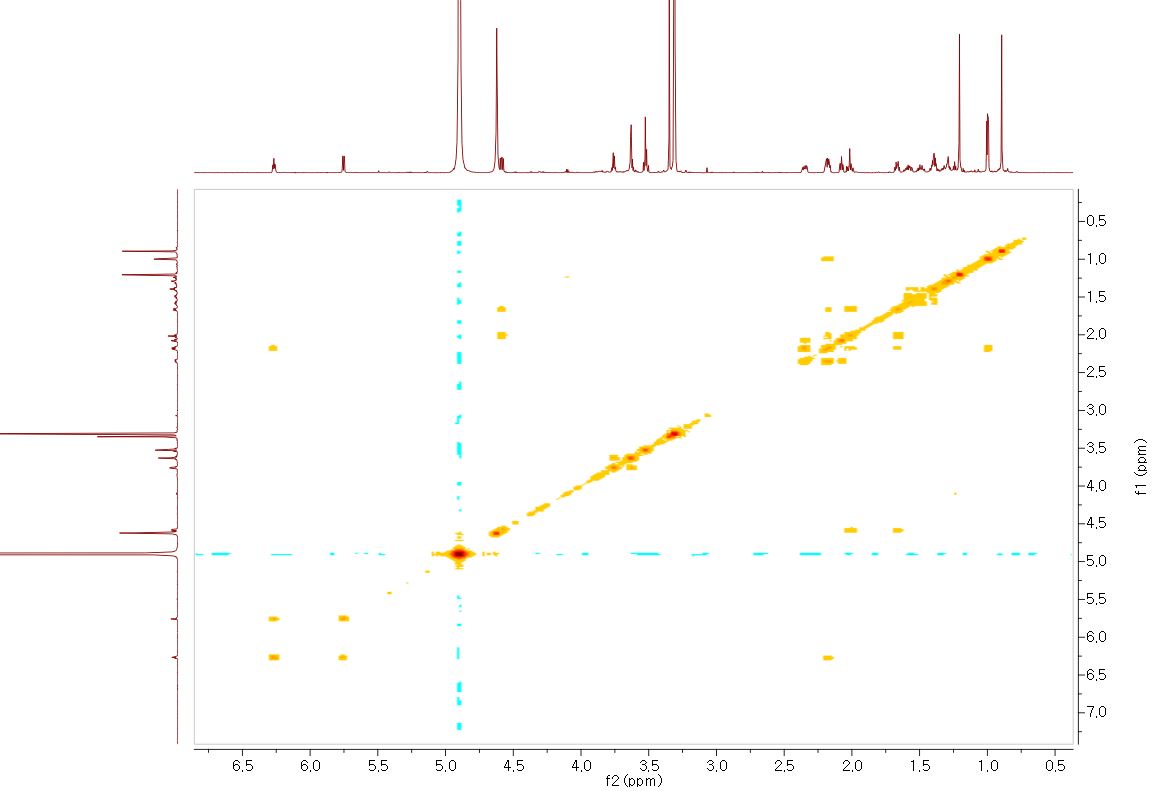


**Figure S16.** The HSQC spectrum of **2**

**
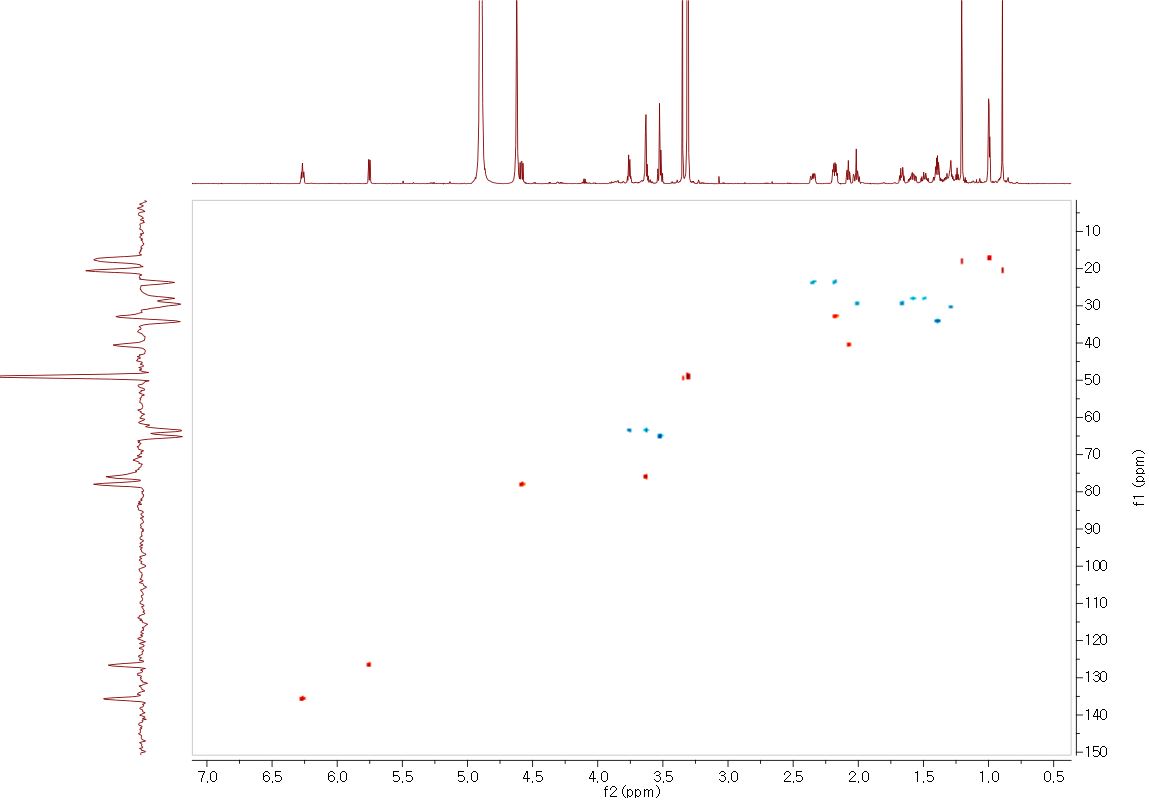
**

**Figure S17.** The HMBC spectrum of **2**


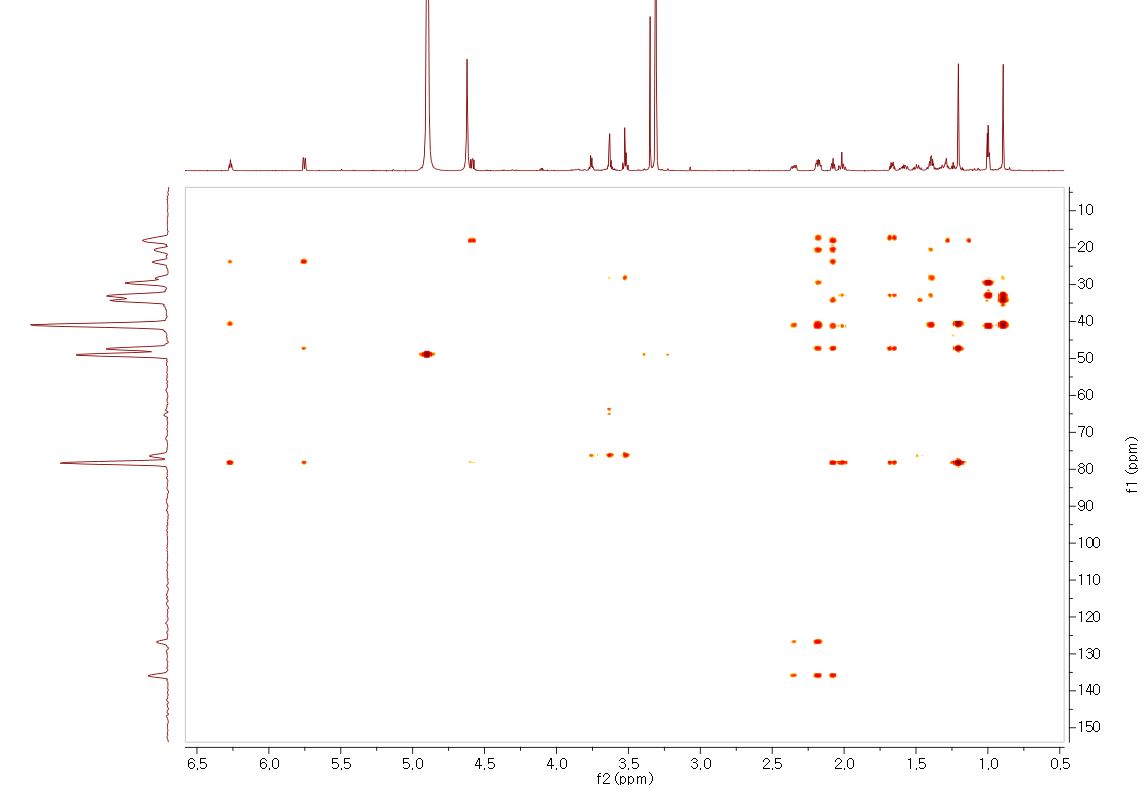


**Figure S18.** The NOESY spectrum of **2**


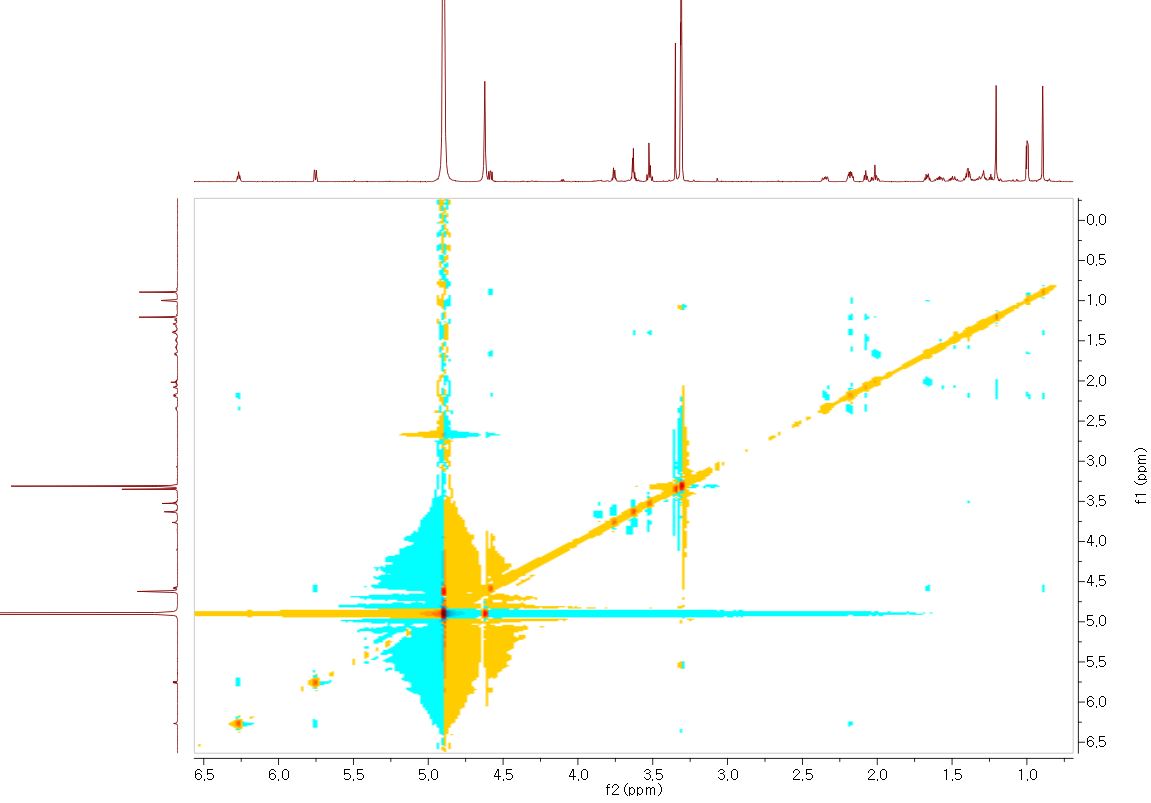


**Figure S19.** 2D NOESY spectrum slice showing irradiation at H-6 in **2**

**
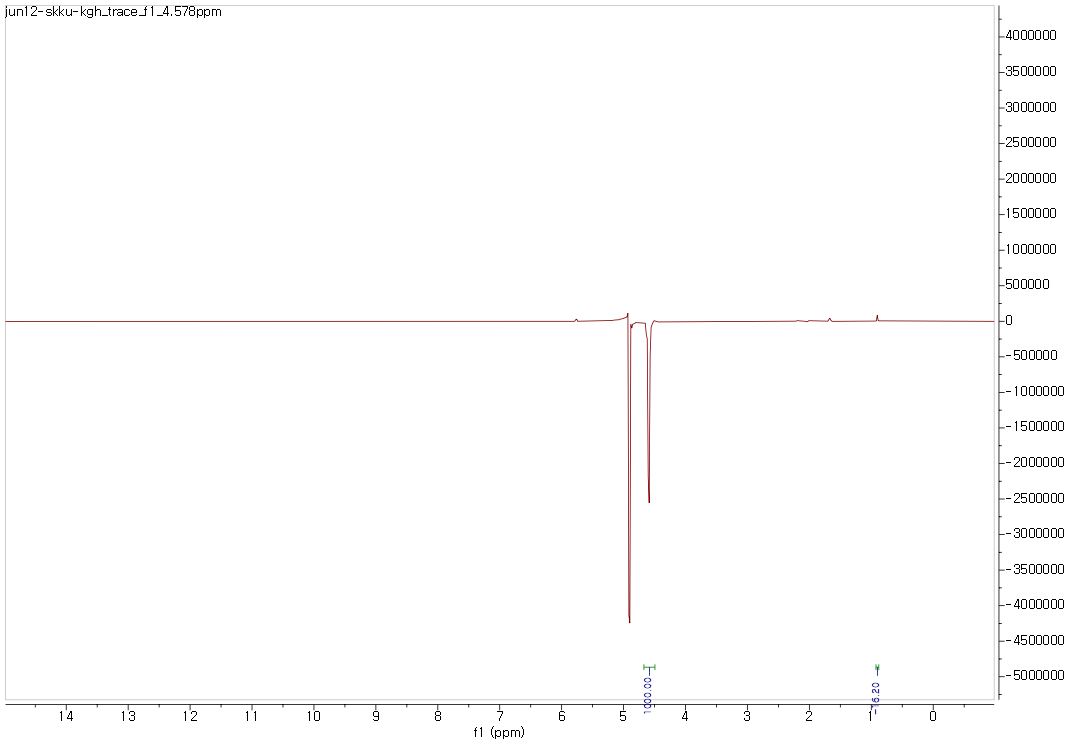
Figure S20.** Calculated Interproton distance between H-6 and H-20 based on the different possible orientation of the 4-OH in compound **2**

H-20

16.20

**
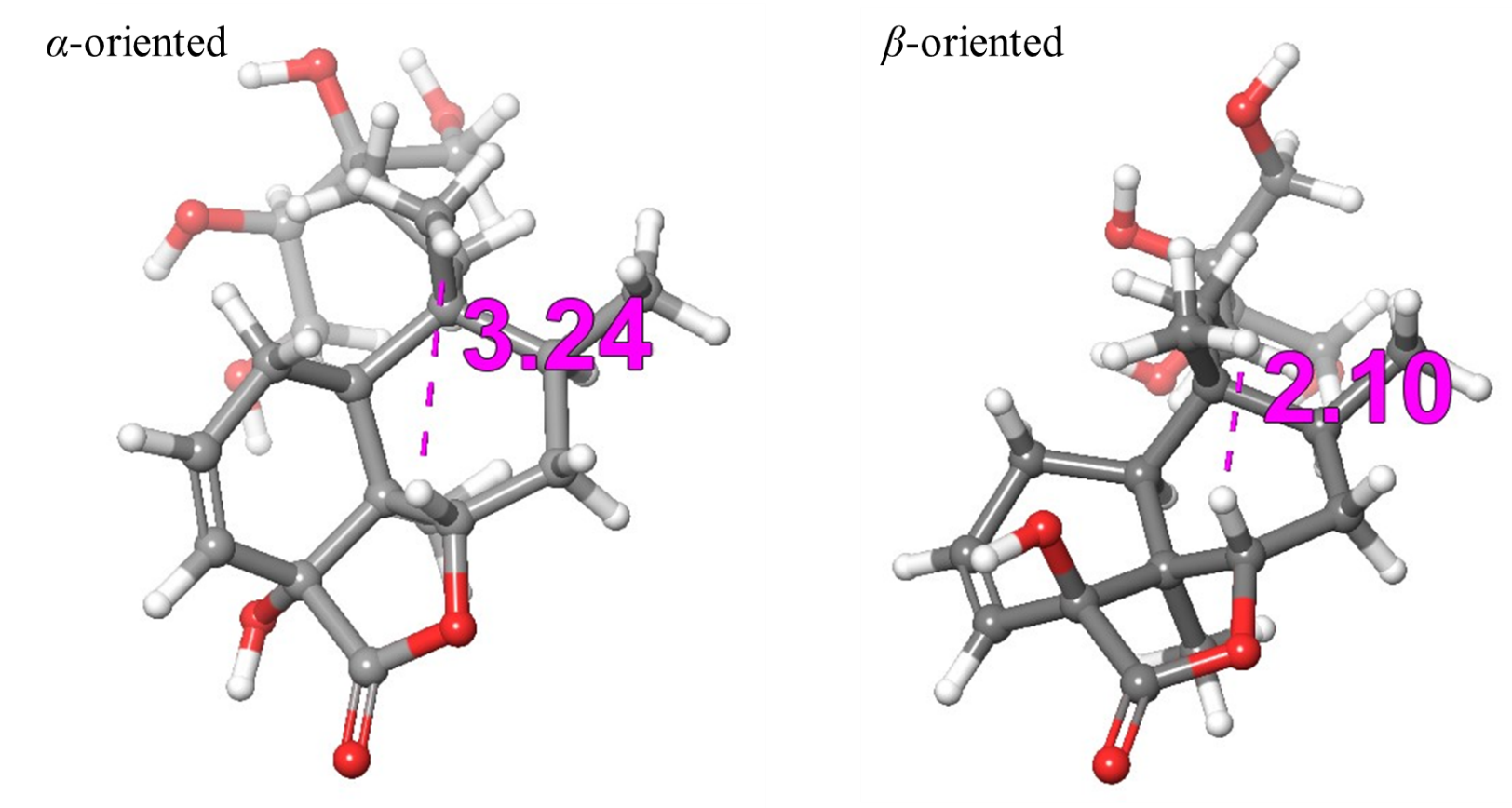
**

**Figure S21.** The DP4+ analysis for **2**

**
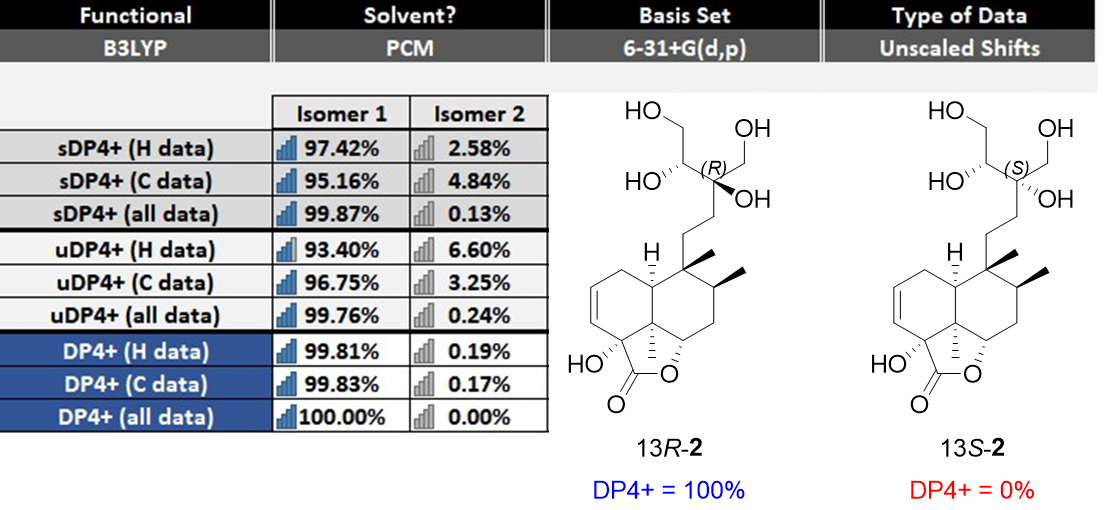
**

**Figure S22.** The HR-ESIMS data of **3**


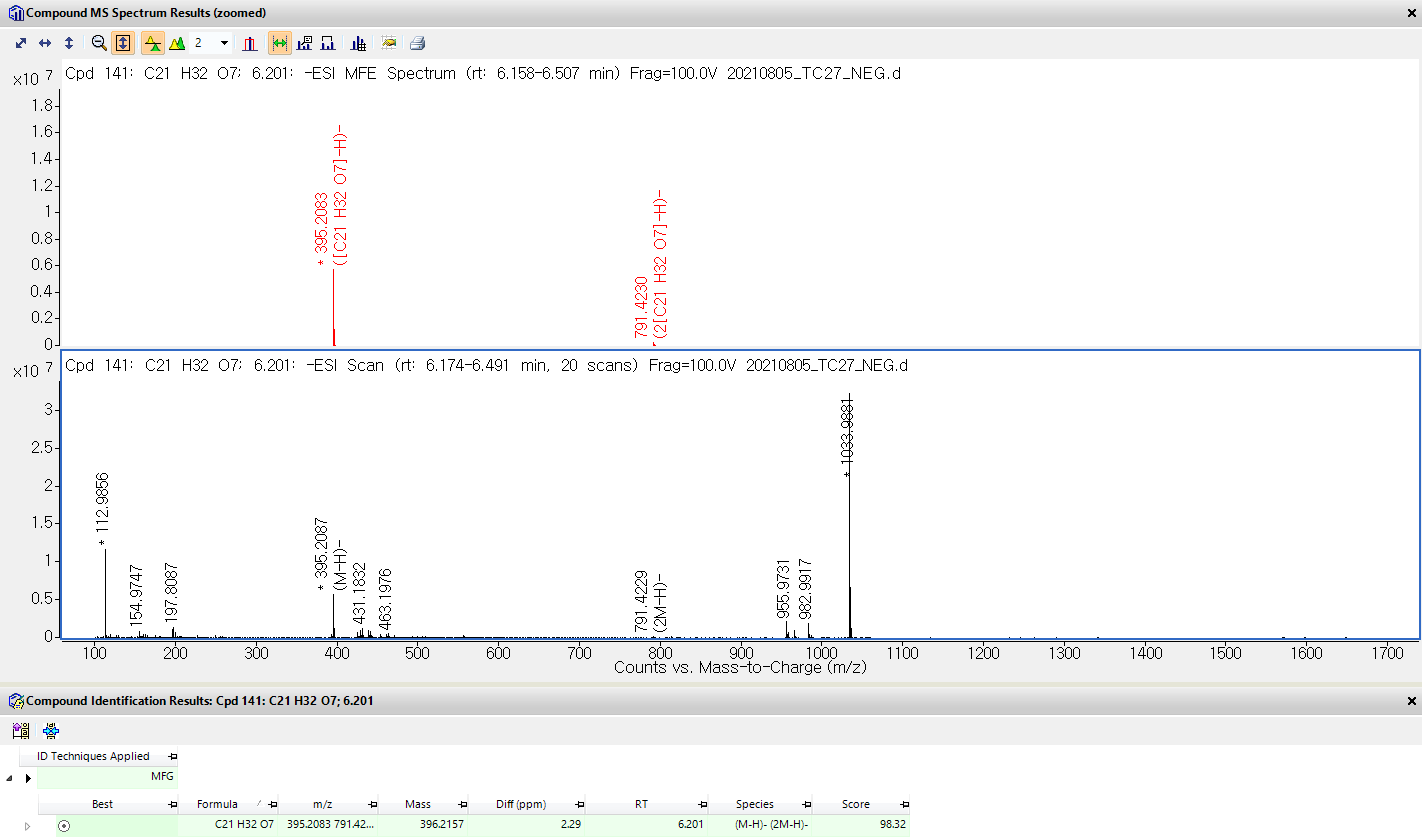


**Figure S23.** The UV spectrum of **3**

**
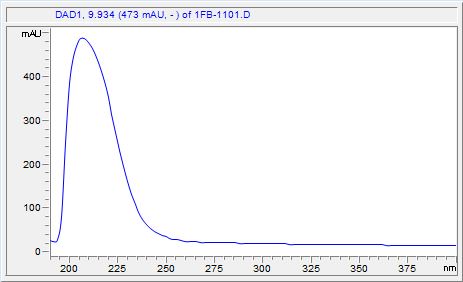
**

**Figure S24.** The ^1^H NMR spectrum of **3** (CD_3_OD, 850 MHz)


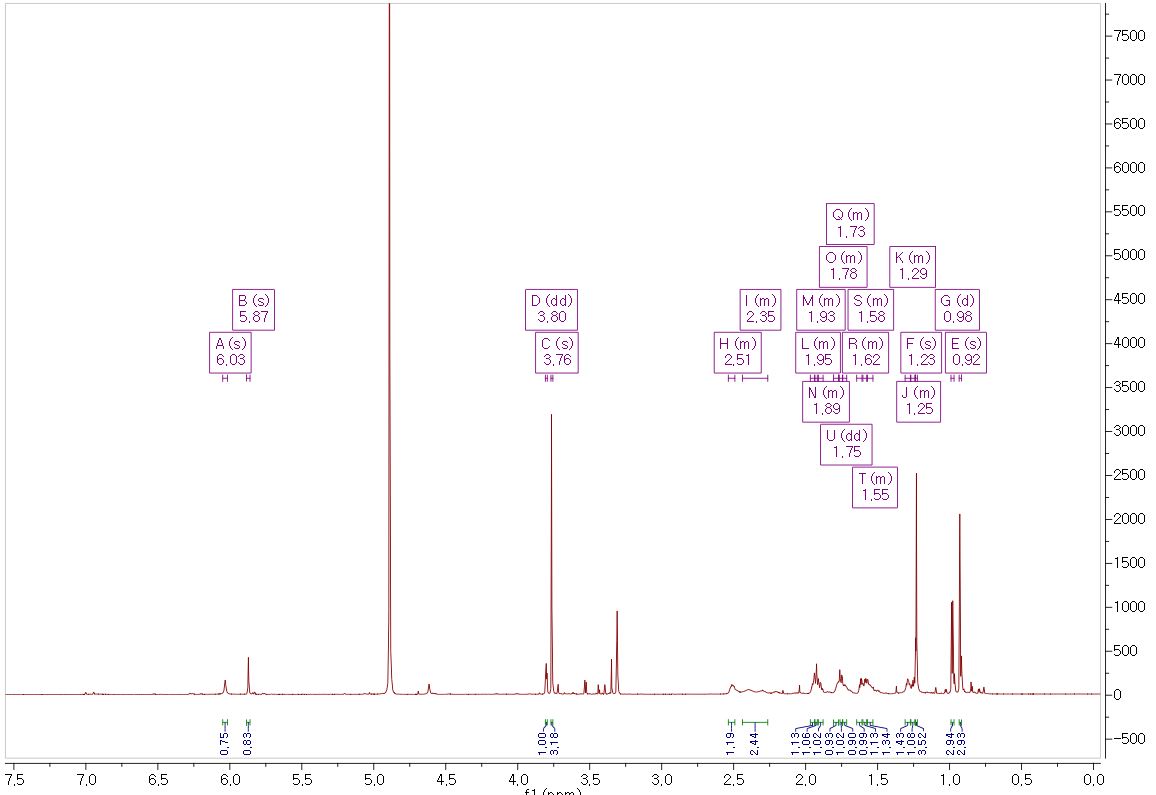


**Figure S25.** The ^1^H-^1^H COSY spectrum of **3**


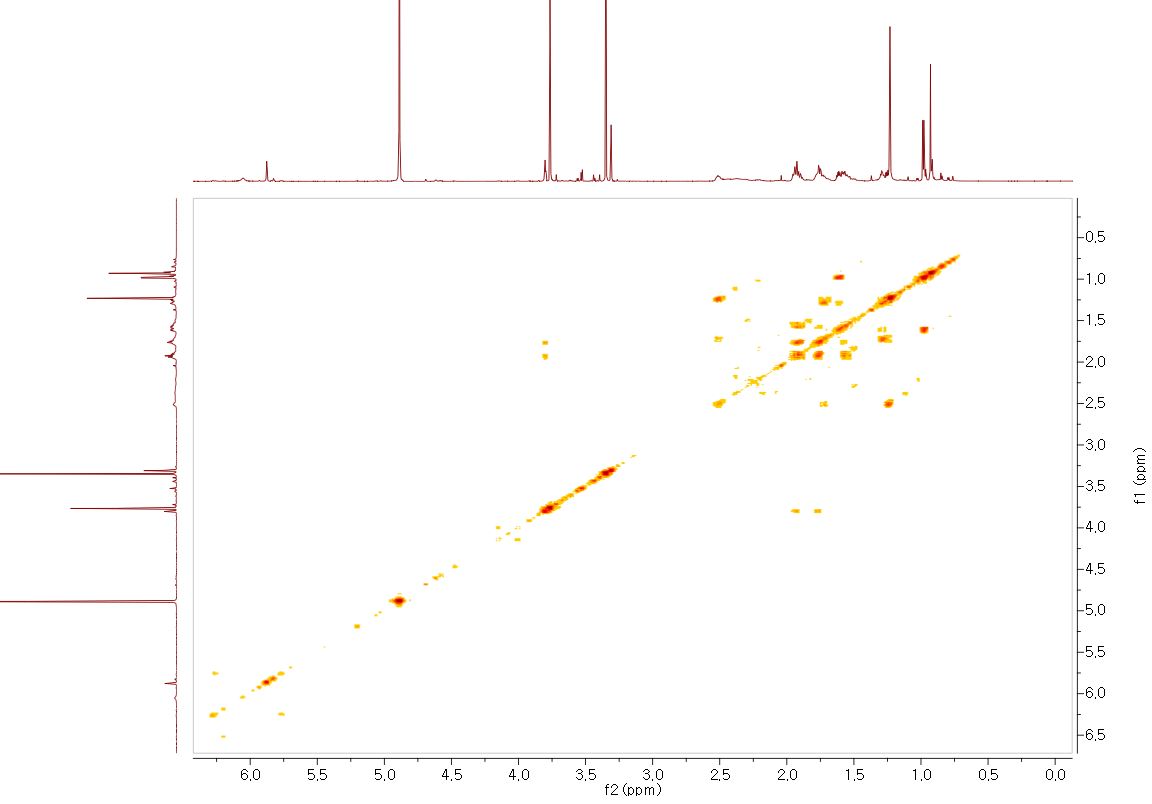


**Figure S26.** The HSQC spectrum of **3**


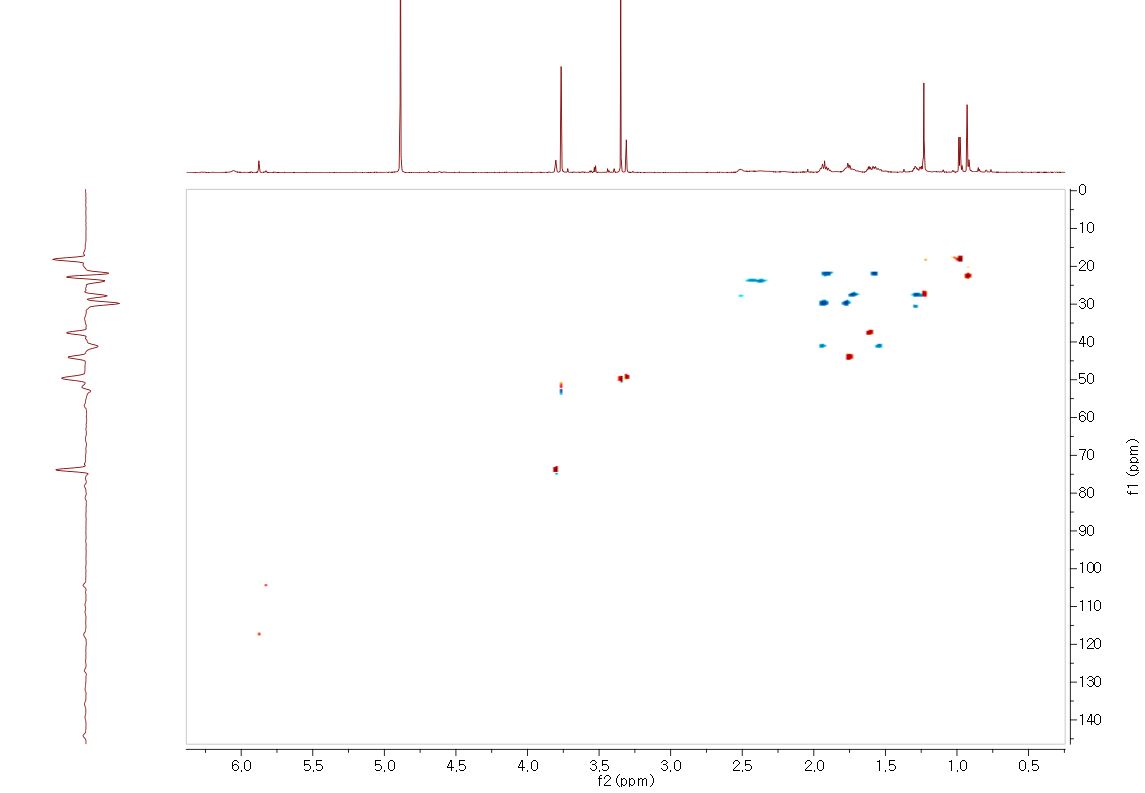


**Figure S27.** The HMBC spectrum of **3**


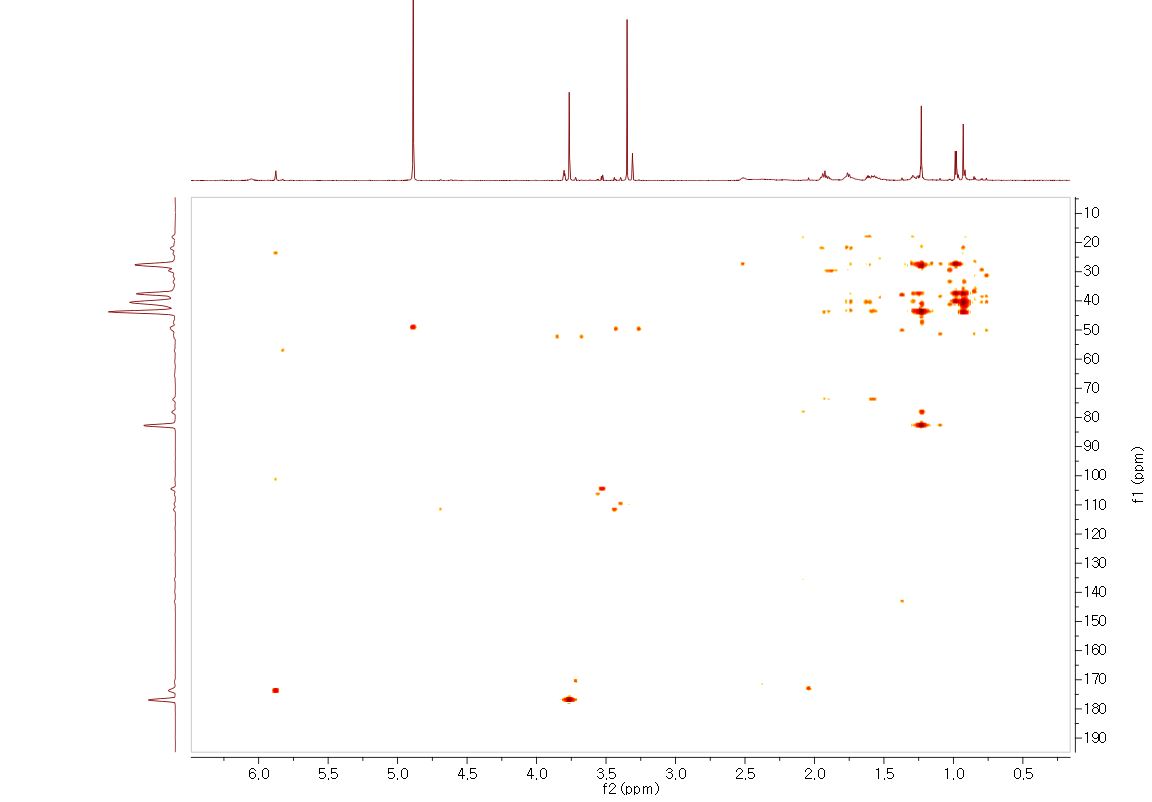


**Figure S28.** The NOESY spectrum of **3**


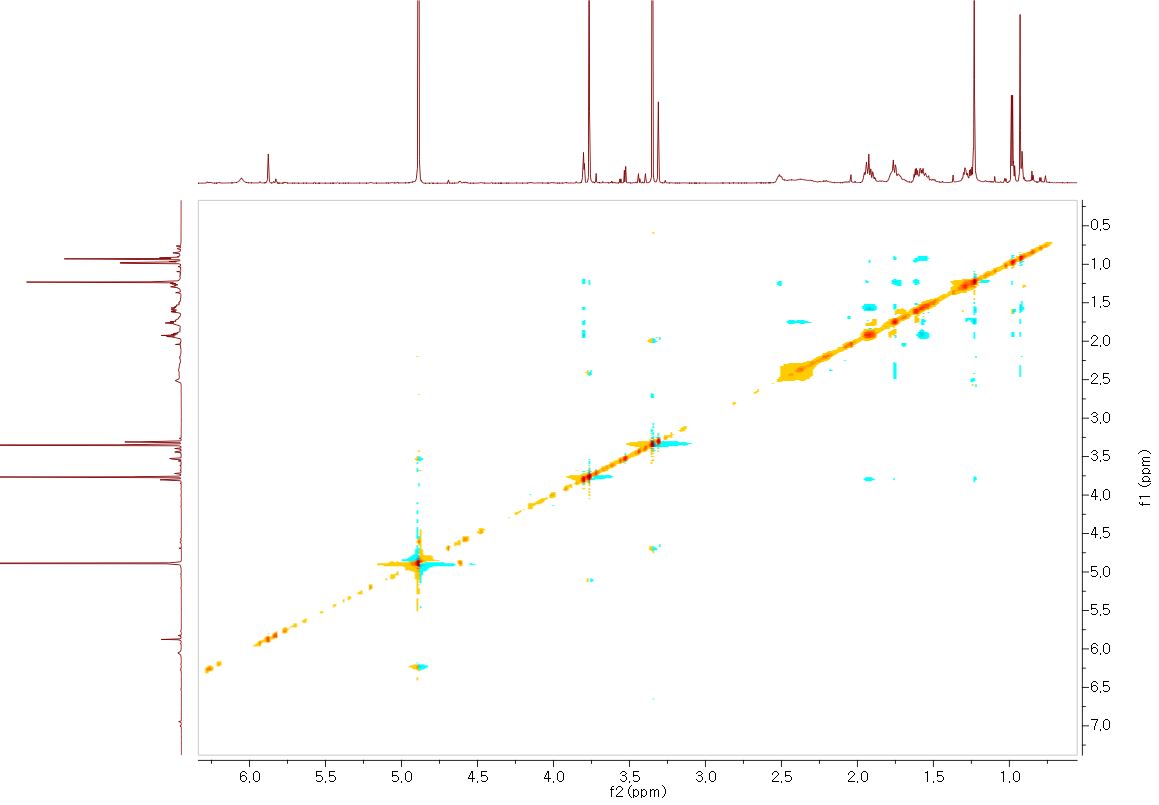


**Figure S29.** The DP4+ analysis for **3**


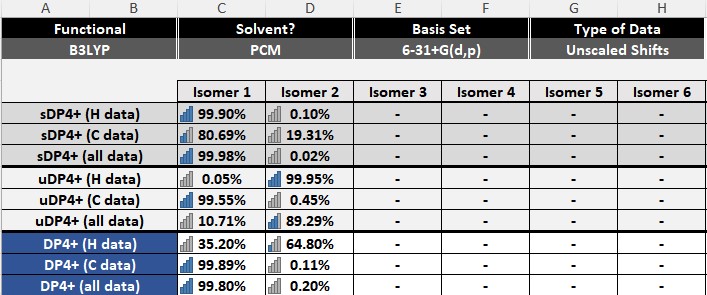


**Figure S30.** The HR-ESI-MS data of **4**


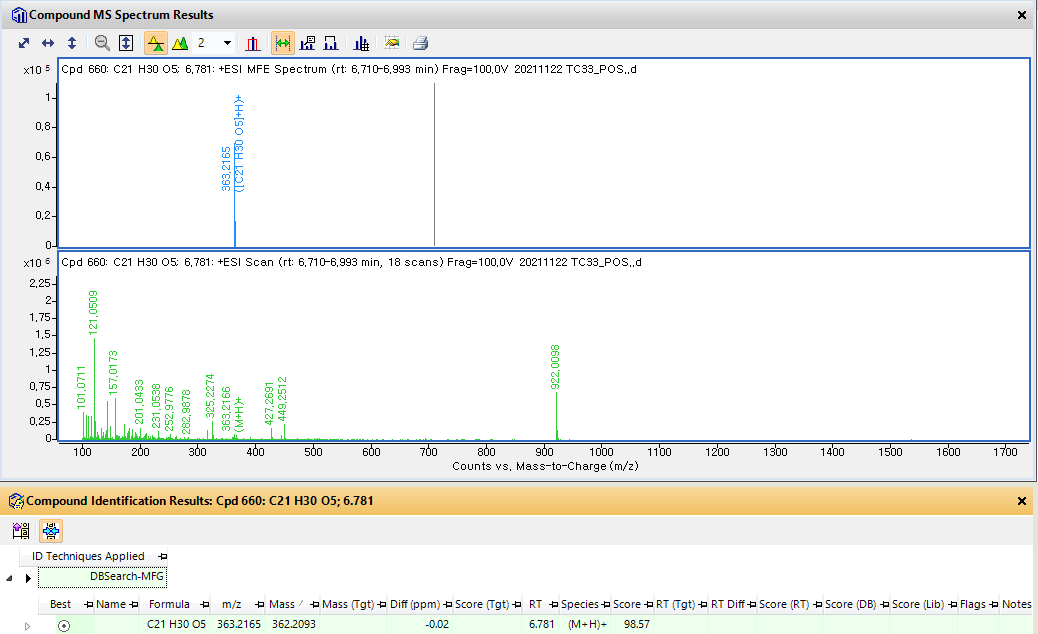


**Figure S31.** The UV spectrum of **4**

**
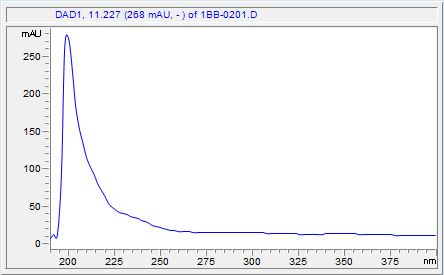
**

**Figure S32.** The ^1^H NMR spectrum of **4** (CD_3_OD, 850 MHz)
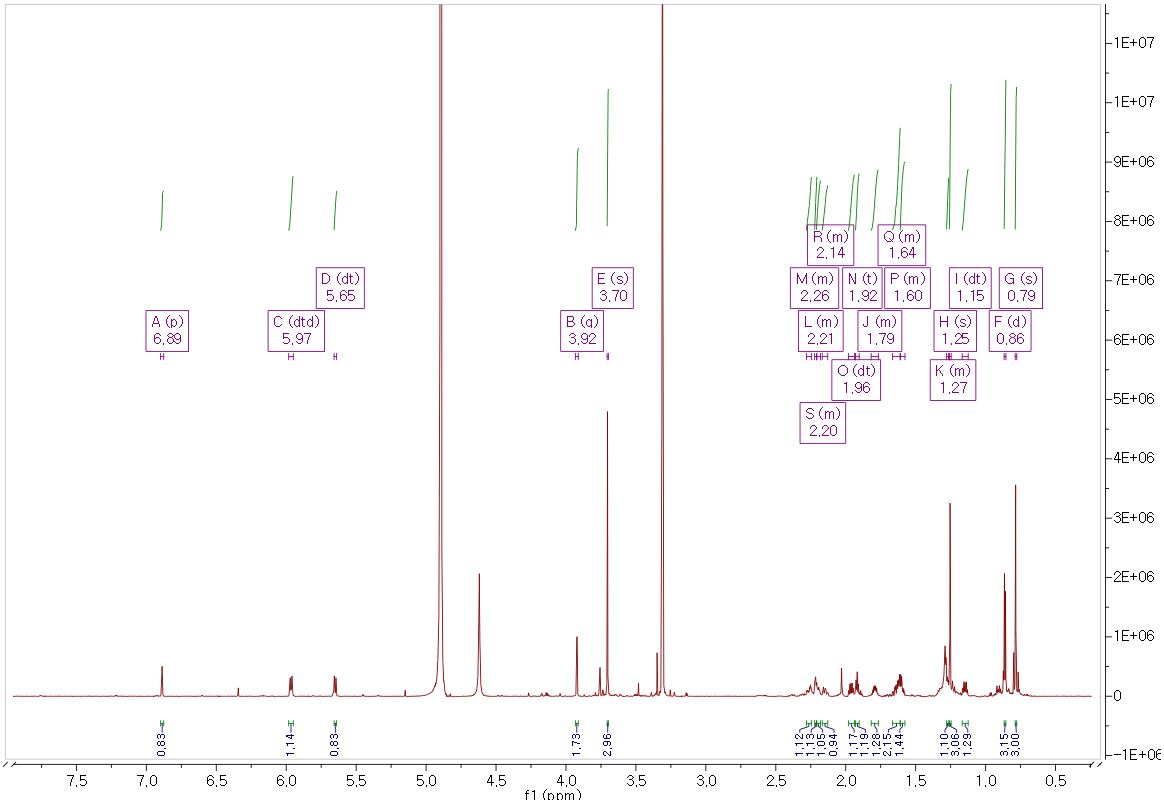


**Figure S33.** The ^13^C NMR spectrum of **4** (CD_3_OD, 212.5 MHz)


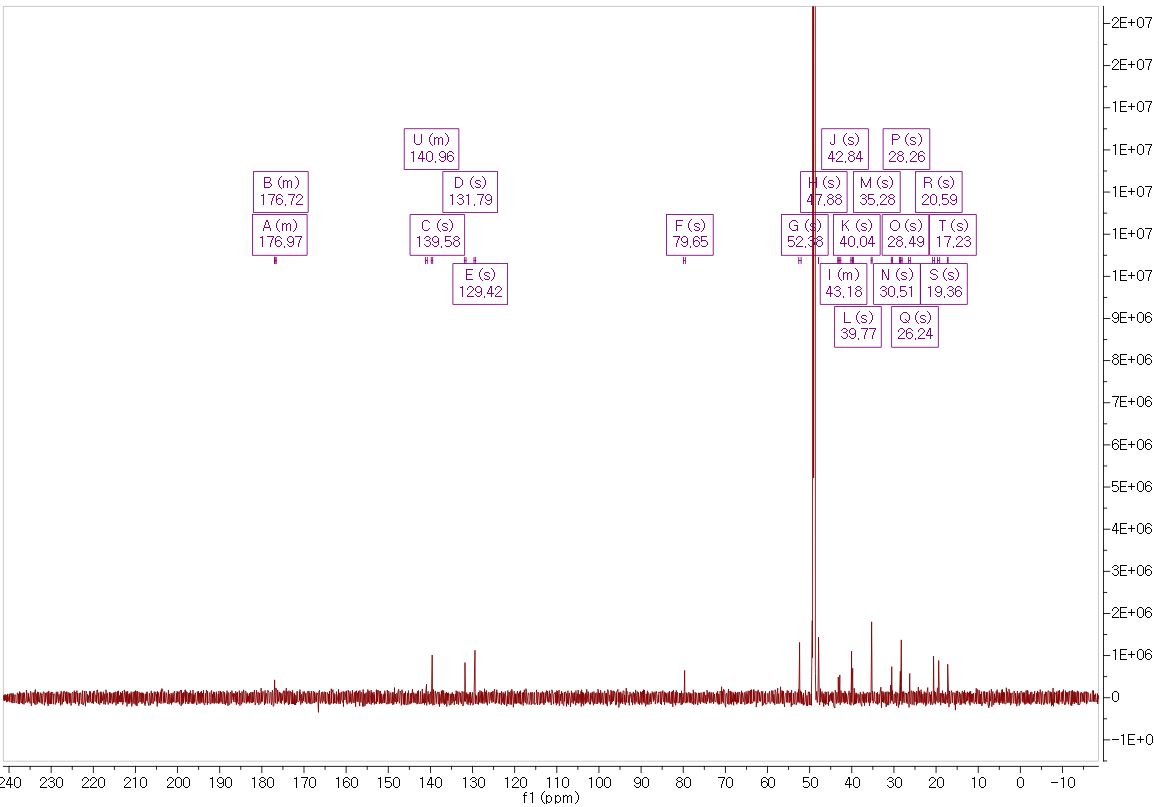


**Figure S34.** The ^1^H-^1^H COSY spectrum of **4**


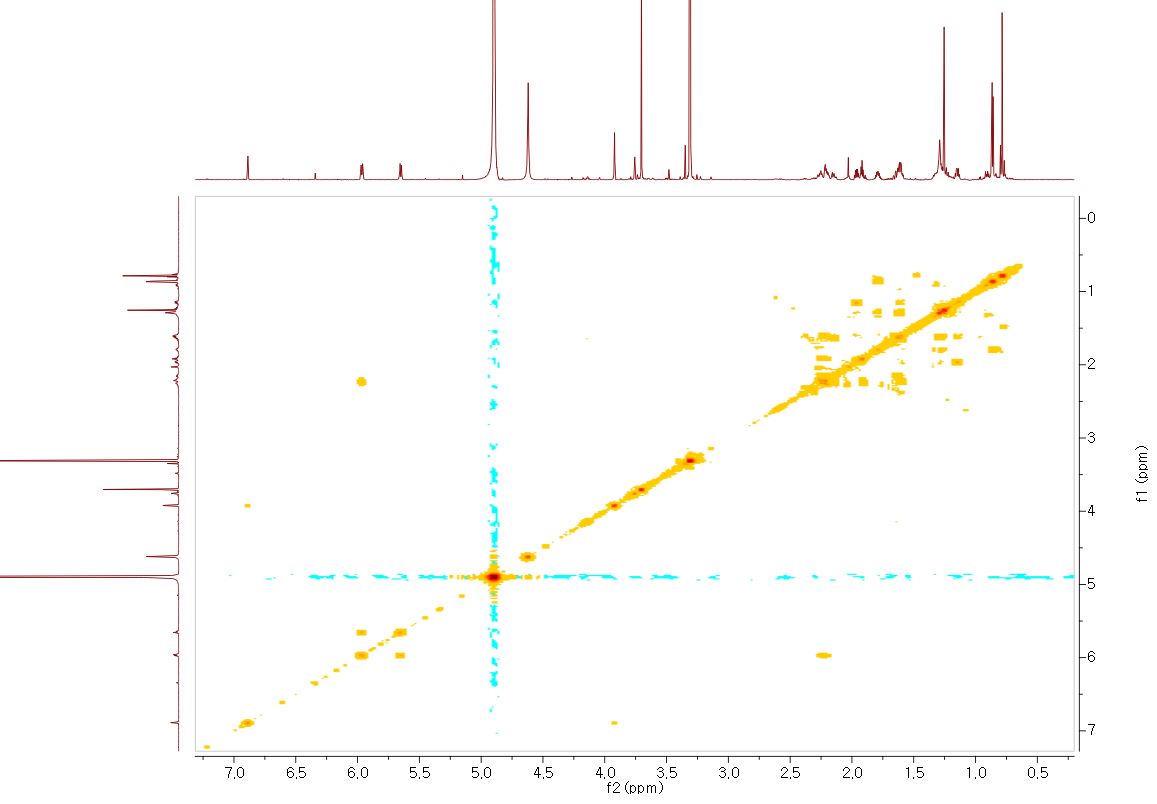


**Figure S35.** The HSQC spectrum of **4**


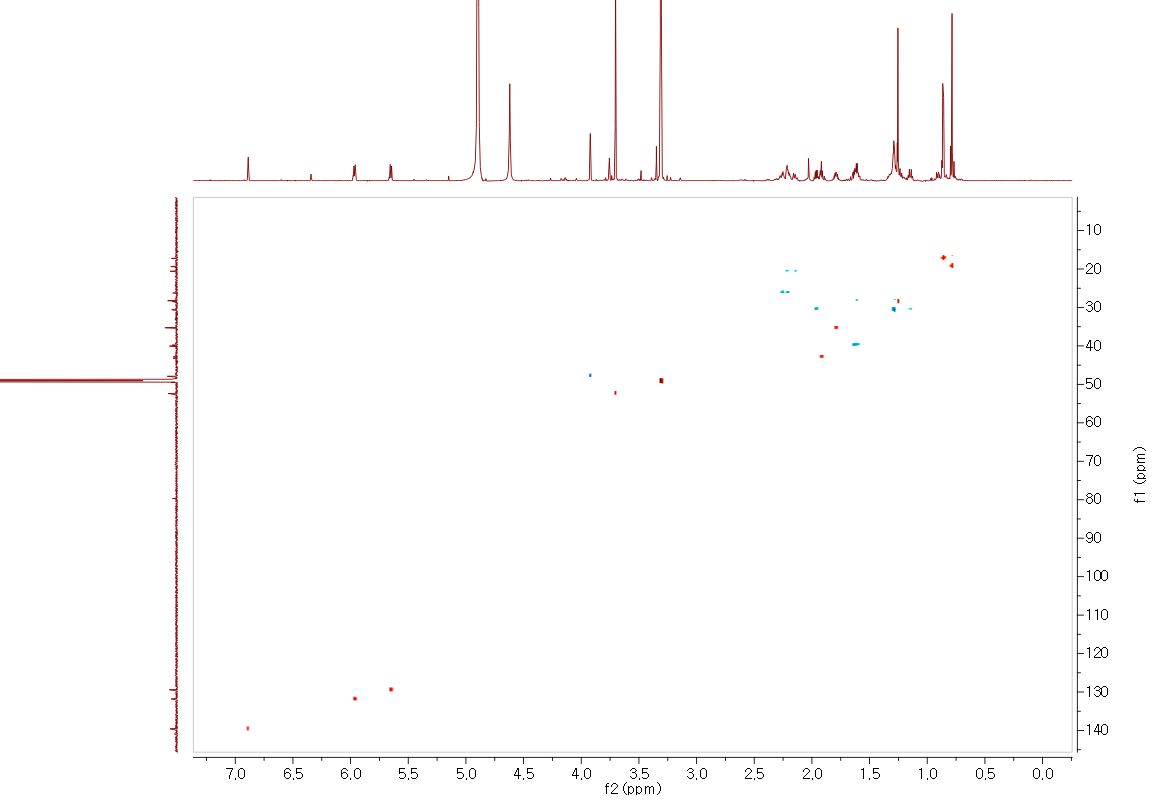


**Figure S36.** The HMBC spectrum of **4**


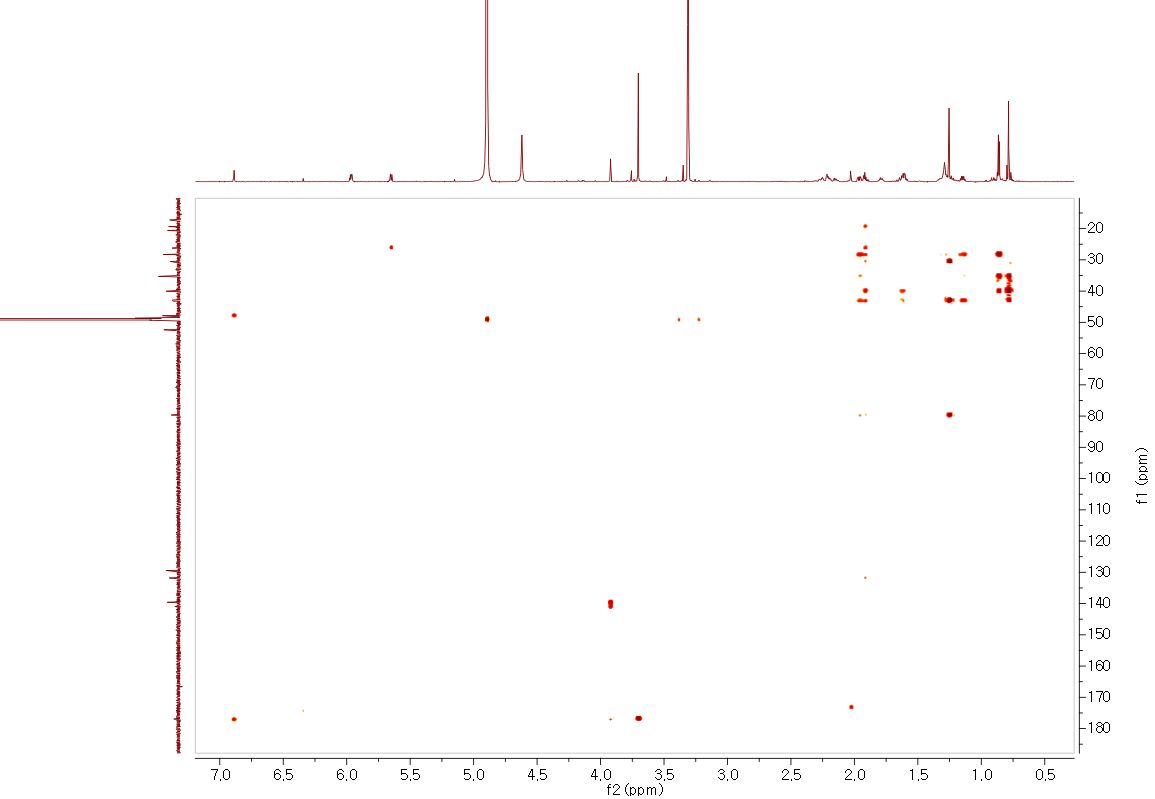


**Figure S37.** The NOESY spectrum of **4**


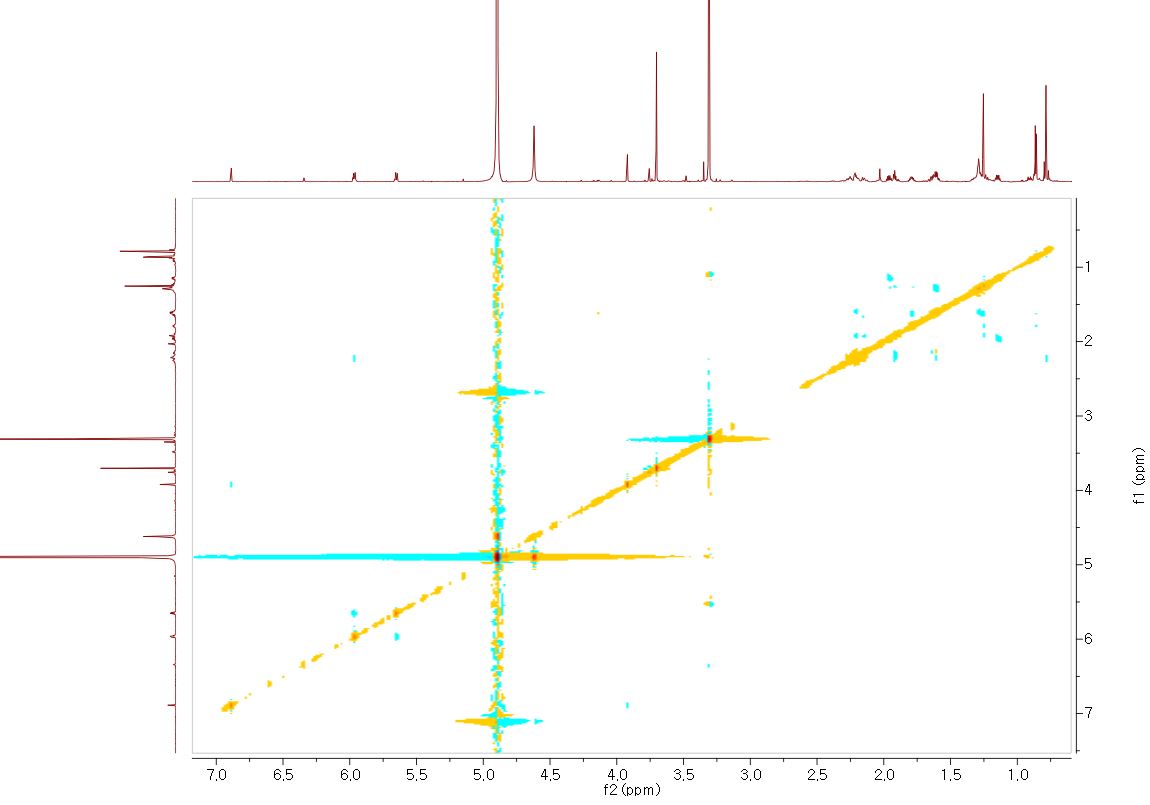


**Figure S38.** The negative ion mode LC/MS data of **5**

**
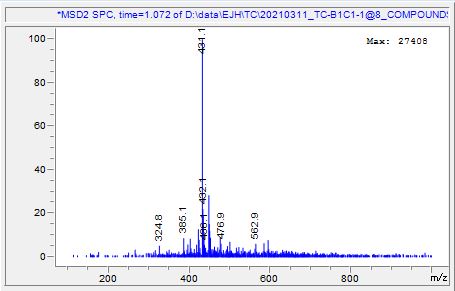
**

**Figure S39.** The ^1^H NMR spectrum of **5** (CD_3_OD, 850 MHz)


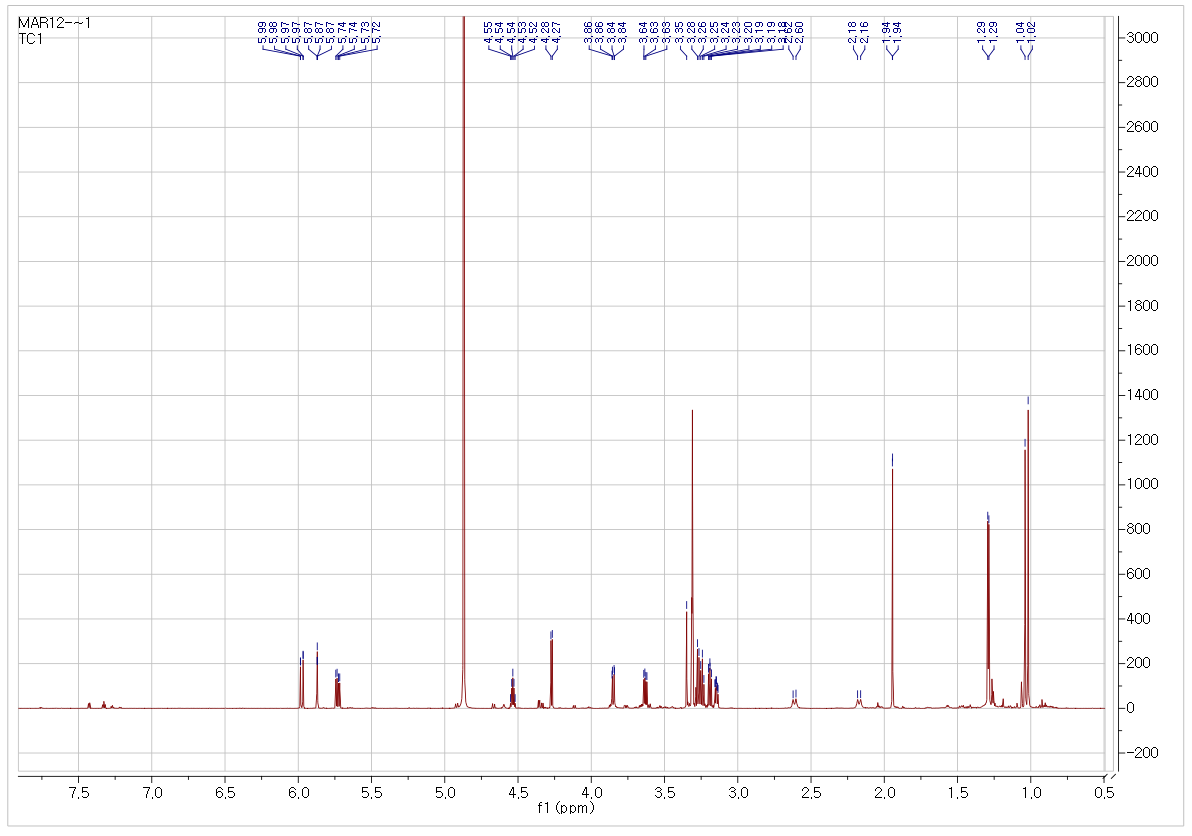


**Figure S40.** Quantification of Western blot band intensities. Band intensities were quantified and normalized to the loading control for each lane. Data are presented as mean ± SEM from three independent experiments (n = 3). Statistical significance was evaluated using a two-tailed unpaired t-test. Significance is indicated as follows: *P < 0.05, **P < 0.01, ***P < 0.001, ****P < 0.0001.

**
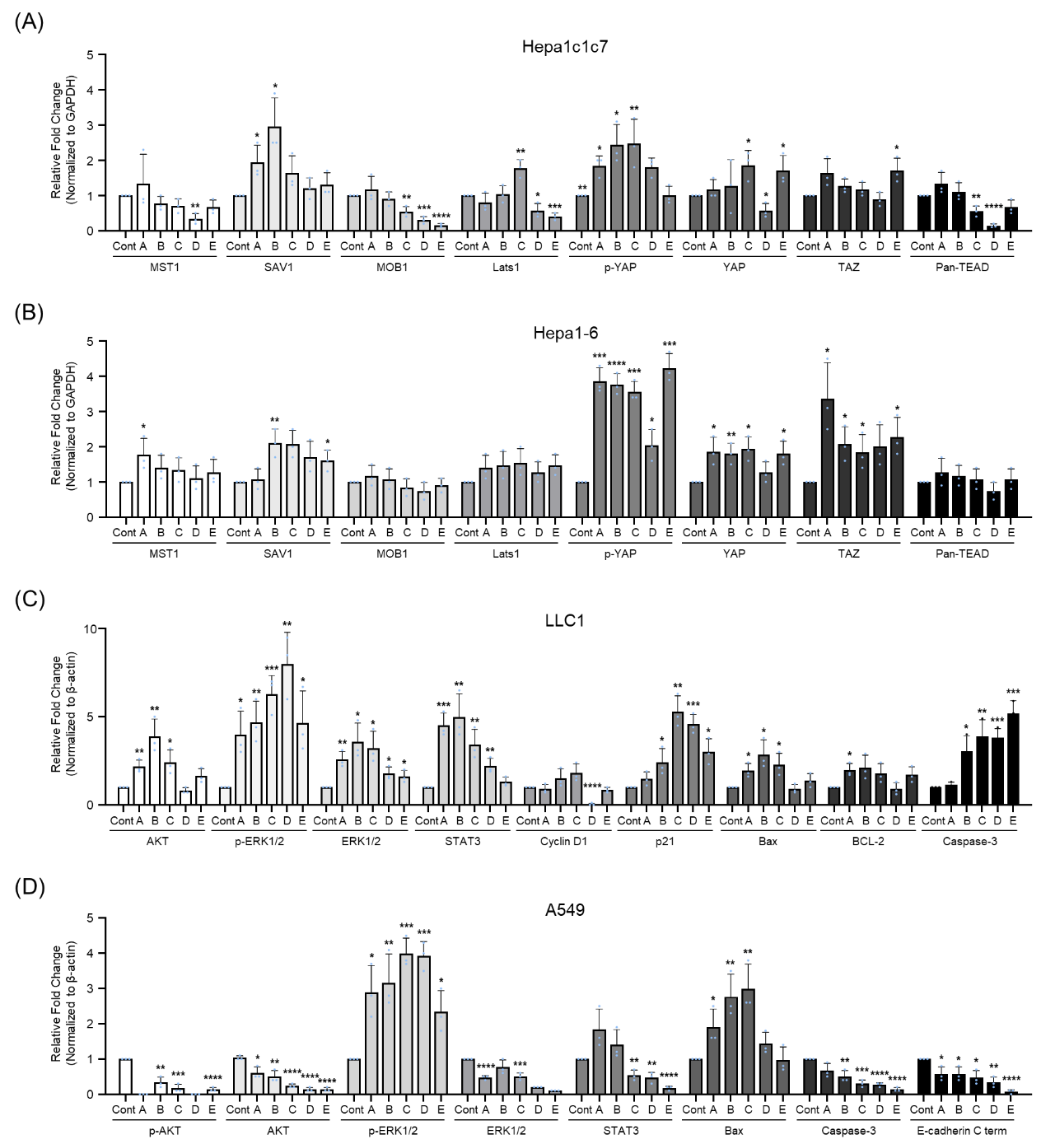
**
